# Integrated metabolomics and antioxidant activity assessment of *Sphagneticola trilobata* (L.) Pruski

**DOI:** 10.1038/s41598-026-54096-w

**Published:** 2026-06-13

**Authors:** Manar T. Ali, Muhammad A. Alsherbiny, Dalia A. Al-Mahdy, Ahlam M. El Fishawy, Asmaa M. Otify

**Affiliations:** 1 Department of Pharmacognosy, Faculty of Pharmacy, Cairo University, Kasr-El-Ainy, Cairo, 11562 Egypt; 2 Department of Pharmacognosy, Faculty of Pharmacy, Modern University for Technology and Information (MTI University), Cairo, Egypt; 3 Innovation Centre, Victor Chang Cardiac Research Institute, Darlinghurst, NSW Australia

**Keywords:** *Sphagneticola trilobata*, Flowerheads, Leaves, UPLC-MS/MS, Antioxidant, Chemometrics, Biochemistry, Biological techniques, Chemistry, Plant sciences

## Abstract

*Sphagneticola trilobata* (L.) Pruski (Asteraceae), formerly known as *Wedelia trilobata*, is a widely distributed ornamental plant utilized in traditional medicine for various ailments. This study aims to provide the first comprehensive comparative analysis of the metabolomic profiles of *Sphagneticola trilobata* flowerheads and leaves to elucidate the chemical basis for their differential antioxidant capacities. Ultra-high-performance liquid chromatography-electrospray ionization-quadrupole time-of-flight mass spectrometry was employed in both positive and negative ionization modes. The resulting datasets were subjected to multivariate chemometric analyses, including Principal Component Analysis and Orthogonal Partial Least Squares Analysis. Antioxidant potential was assessed using in vitro DPPH, FRAP, and ABTS assays. A total of 86 metabolites were tentatively annotated, spanning classes of terpenoids, flavonoids, phenolic acids, lipids, and amino acids. Notably, 18 of these compounds were observed in this plant for the first time. Chemometric analysis revealed distinct metabolic variances: flowerheads were enriched with flavonoids and phenolic acids, whereas leaves were characterized by a predominance of sesquiterpenoids and diterpenoids. Biological assays demonstrated that flowerheads possess significantly higher antioxidant activity compared to leaves. Moreover, correlation analysis identified key biomarkers responsible for this activity, including 3’,4’,7-trihydroxy-flavanone hexoside, luteolin, myricetin hexoside, and 4,5-dicaffeoyl-quinic acid. The study establishes that the superior antioxidant potential of *Sphagneticola trilobata* flowerheads is driven by their high flavonoid and phenolic acid content, distinguishing them from the terpenoid-rich leaves. These findings validate the utility of metabolomics in identifying bioactive markers and suggest the flowerheads as a promising source of natural antioxidants for pharmaceutical or cosmeceutical applications.

**Supplementary Information:**

The online version contains supplementary material available at 10.1038/s41598-026-54096-w.

## Introduction

*Sphagneticola trilobata* (*S. trilobata*), formerly known as *Wedelia trilobata*, is a creeping plant of the Asteraceae family native to Central America but now invasive in regions like China, Florida, and South Africa^1^. Beyond its ornamental value, it holds a significant place in traditional medicine, including Ayurveda and Traditional Chinese Medicine, for treating skin inflammation, wounds, liver dysfunction, infertility, cold, and cough. Flowerheads and leaves were highly valued in traditional medicine due to their ability to relieve many disorders. In detail, syrups of leaves and flowerheads were employed to alleviate cough and fever in India. Poultices of crushed leaves were applied topically to treat injured skin and painful joints. Also, Flowerheads were used to treat symptoms of diabetes^2^.

Owing to the plant’s significance in traditional medicine, extensive research has been conducted to characterize phytochemical constituents and evaluate the biological potentials of the plant, with studies concentrating on the leaves and flowerheads. Notably, the plant is enriched with different terpenoids, especially sesquiterpenoids and diterpenoids, and flavonoids. Several compounds were isolated from flowerheads and leaves, such as kaurenoic acid (KA), grandifloric acid, grandiflorenic acid, stigmasterol, luteolin, kaempferol, and rutin, however, no comprehensive metabolomic study has yet been conducted to identify the phytochemical profile of each organ^2^.

Regarding the biological profile, studies confirm that the leaves promote wound-healing and possess antibacterial, anthelmintic, antimalarial, antivenom, and cytotoxic properties, whereas the flowerheads exhibit significant anti-inflammatory, antimicrobial, and antidiabetic activities. Moreover, various isolated metabolites demonstrated biological effects, namely, anti-inflammatory, hepatoprotective, and antimicrobial potentials of KA, wound healing impact of grandiflorenic acid, and cytotoxic effect of trilobolide-6-isobutyrate^2^. It is worth noting that these studies examined only the biological effects of the isolated compounds, underscoring the importance of the metabolic profile in revealing additional metabolites that may be more active.

A common thread linking these diverse biological benefits is the plant’s powerful antioxidant capacity, which helps combat oxidative stress, a fundamental cause of chronic diseases. However, the reported antioxidant potency varies considerably, with some studies indicating flowerheads are more potent, while others find them less powerful than leaves^3,4^. This inconsistency underscores the influence of extrinsic factors like geography, harvest time, and processing techniques on the plant’s phytochemical makeup. To resolve these inconsistencies and provide a scientific basis for its traditional uses, a comprehensive comparative metabolomic study is essential.

Therefore, this study aims to conduct a comparative analysis of the metabolomic profiles of *S. trilobata* flowerheads and leaves and correlate these profiles with their antioxidant activities. Ultra-high-performance liquid chromatography-electrospray ionization-quadrupole time-of-flight mass spectrometry (UPLC-ESI-QTOF-MS/MS), coupled with multivariate data analysis, was employed to elucidate the specific phytochemicals responsible for the observed bioactivities.

## Results and discussion

### UPLC-ESI-QTOF-MS/MS metabolite profiling

**Fig. 1:**
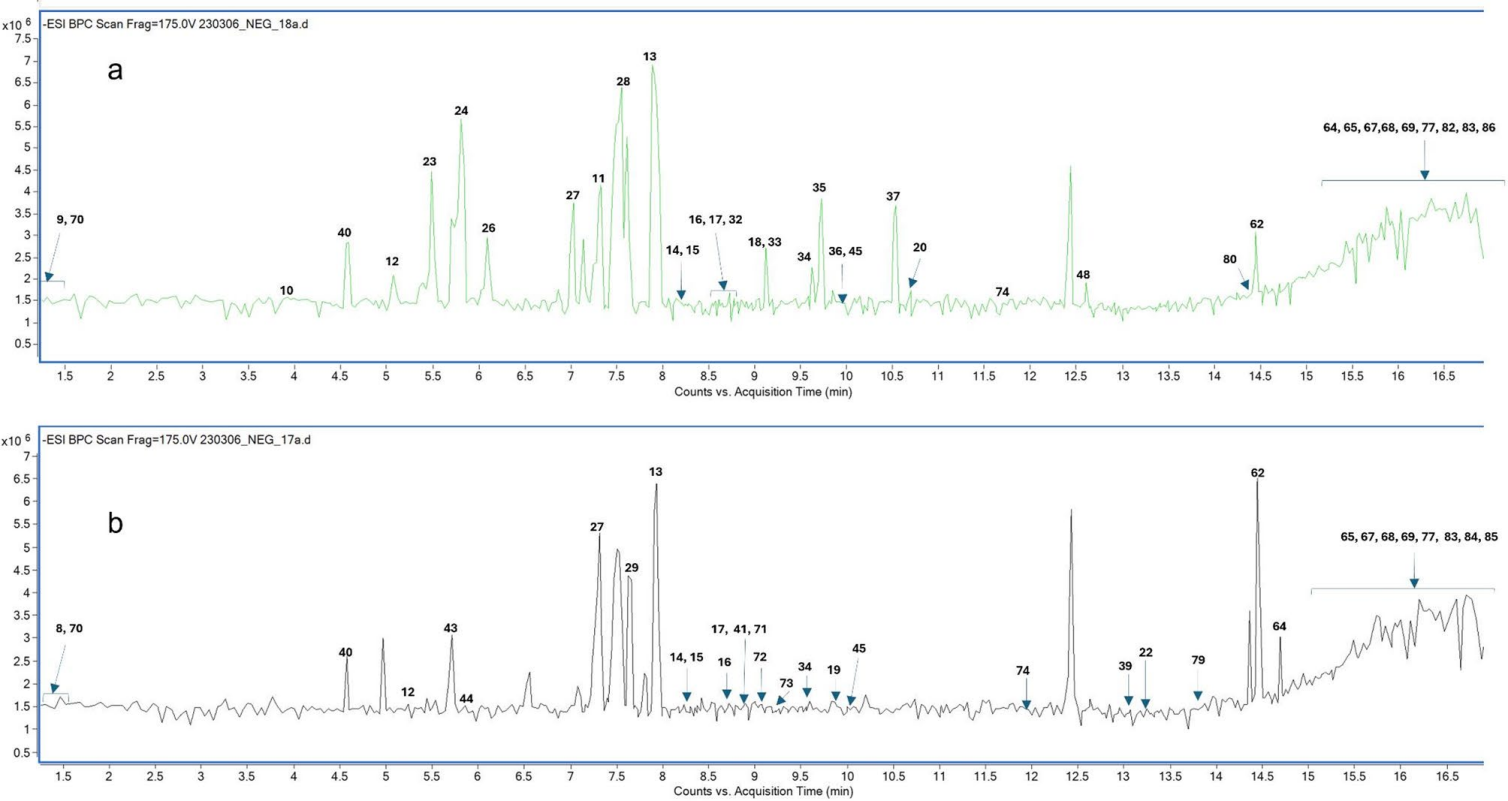
UPLC base peak chromatograms of *S. trilobata* **(a)** flowerheads and **(b)** leaves extracts in the negative ion mode. Peak ID follows that listed in Table 1.

**Fig. 2:**
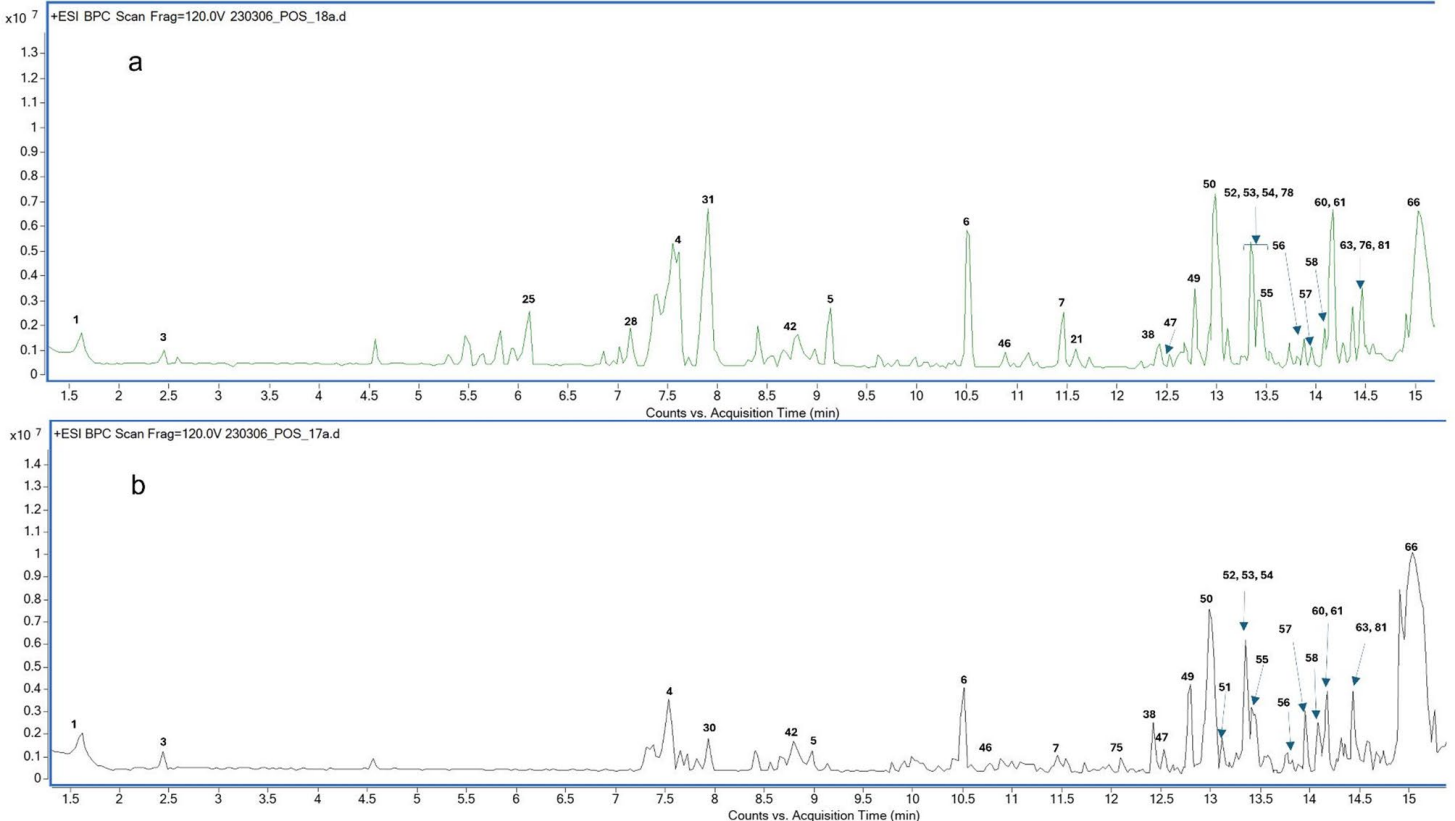
UPLC base peak chromatograms of *S. trilobata* (**a)**. flowerheads and (**b).** leaves extracts in the positive ion mode. Peak ID follows that listed in Table 1.

Metabolomic analysis revealed that *S. trilobata* is enriched with diverse compounds such as terpenoids, flavonoids, and phenolic acids. A total of 86 metabolites were tentatively annotated, belonging to different chemical classes, namely, 27 terpenoids, 17 flavonoids, 13 phenolic acids, 9 lipids, 8 carboxylic and fatty acids derivatives, 7 amino acids, 3 other aromatic compounds, and 2 carbohydrates as shown in Table 1 and in the UPLC base peak chromatograms for the ethanolic extracts of flowerheads and leaves, obtained in negative and positive modes Figs. 1 and 2.

**Table 1:** Tentatively annotated metabolites in *S. trilobata* flowerheads and leaves ethanolic extracts by UPLC-ESI-QTOF-MS/MS in negative and positive ion modes. FA, formic acid; F, Major metabolite in flowerheads; L, Major metabolite in leaves; S, S-plotted differential metabolites; numbers in bold represent the base peak.

ID	*R*_t_ (min)	Measured m/z	Adduct	Error (ppm)	Molecular formula	MS/MS fragments	Compound name	Tags #	References
**Amino acids**									
**1**	1.40	146.1177	[M + H]^+^	−1	**C**_**7**_ **H**_**15**_ **NO**_**2**_	**118**, 59, 58	Homonorleucine	F, L	HMDB
**2**	1.16	116.0718	[M–H]^–^	−0.84	**C**_**5**_ **H**_**11**_ **NO**_**2**_	83, 64, **58**	Valine		HMDB
	1.45	118.0864	[M + H]^+^	−1.24	**C**_**5**_ **H**_**11**_ **NO**_**2**_	72, 59, **58**			HMDB
**3**	2.56	166.0863	[M + H]^+^	−0.27	**C**_**9**_ **H**_**11**_ **NO**_**2**_	**120**, 103, 77	L-Phenylalanine	F, L	^36^
	2.63	164.0718	[M–H]^–^	−0.59	**C**_**9**_ **H**_**11**_ **NO**_**2**_	**147**, 120, 103			HMDB
**4**	7.71	480.2598	[M + H]^+^	−1.27	**C**_**25**_ **H**_**37**_ **NO**_**8**_	**128**	Pyroglutamic acid derivative	F, L	
**5**	9.13	508.2553	[M + H]^+^	0.29	**C**_**27**_ **H**_**33**_ **N**_**5**_ **O**_**5**_	**128**	Pyroglutamic acid derivative	S, F	
**6**	10.51	568.2778	[M + H]^+^	−2.16	**C**_**29**_ **H**_**37**_ **N**_**5**_ **O**_**7**_	550, 522, **118**	Valine derivative	S, F, L	
**7**	11.46	464.2652	[M + H]^+^	0.38	**C**_**25**_ **H**_**39**_ **NO**_**7**_	446, **128**, 100	Pyroglutamic acid derivative	S, F, L	
**Carbohydrates**									
**8**	0.73	539.1383	[M + Cl]^–^	0.25	**C**_**18**_ **H**_**32**_ **O**_**16**_	**503**, 323, 179, 161, 89, 59	Trisaccharide (Raffinose)	L	^36^
**9**	0.75	341.1089	[M–H]^–^	0.1	**C**_**12**_ **H**_**22**_ **O**_**11**_	179, 161, 89, 71, **59**	Disaccharide	F	^37^
**Phenolic acids**									
**10**	4.04	137.0249	[M–H]^–^	−3.49	**C**_**7**_ **H**_**6**_ **O**_**3**_	**137**, 136, 108, 93, 92	*p-*Hydroxybenzoic acid	F	^36^
**11**	7.44	513.0700	[M–H]^–^	1.62	**C**_**21**_ **H**_**22**_ **O**_**13**_ **S**	353, **271**, 191, 135, 97	Caffeoyl-quinic acid sulfate derivative	F	
**12**	5.41	515.1220	[M–H]^–^	−4.84	**C**_**25**_ **H**_**24**_ **O**_**12**_	353, **191**, 179, 135	3,5-Dicaffeoyl-quinic acid	S, F, L	^13^
**13**	7.88	515.1219	[M–H]^–^	−4.65	**C**_**25**_ **H**_**24**_ **O**_**12**_	353, 191, 179, **173**	4,5-Dicaffeoyl-quinic acid	S, F, L	^13^
**14**	8.25	529.1370	[M–H]^–^	−3.49	**C**_**26**_ **H**_**26**_ **O**_**12**_	367, 335, 193, 179, **173**	3- Feruloyl-4-caffeoyl-quinic acid	F, L	^13^
**15**	8.32	337.0941	[M–H]^–^	−3.58	**C**_**16**_ **H**_**18**_ **O**_**8**_	191, **173**, 119	4-Coumaroyl-quinic acid	F	^13^
**16**	8.65	367.1048	[M–H]^–^	−3.65	**C**_**17**_ **H**_**20**_ **O**_**9**_	193, 191, **173**, 134	4-Feruloyl-quinic acid	F, L	^13^
**17**	8.79	499.1245	[M–H]^–^	0.17	**C**_**25**_ **H**_**24**_ **O**_**11**_	353, 191, 179, **173**, 163, 119	Coumaroyl-caffeoyl-quinic acid	F, L	^38^
**18**	9.03	529.1370	[M–H]^–^	−3.49	**C**_**26**_ **H**_**26**_ **O**_**12**_	367, 193, **173**	4-Feruloyl-5-caffeoyl-quinic acid	F, L	^13^
**19**	9.90	441.2493	[M + FA–H]^–^	0.21	**C**_**22**_ **H**_**36**_ **O**_**6**_	441, 395, 351, 335, 163, **59**	Coumaric acid derivative	S, L	^39^
**20**	10.59	691.1663	[M–H]^–^	0.79	**C**_**35**_ **H**_**32**_ **O**_**15**_	**529**, 353, 193, 179, 173	4,5-Dicaffeoyl-3-feruloyl-quinic acid	F	^13^
**21**	11.60	787.3677	[M + H]^+^	3.1	**C**_**46**_ **H**_**50**_ **N**_**4**_ **O**_**8**_	**641**, 495, 275	Tetra coumaroyl spermine	S, F	^14^
**22**	13.35	491.2862	[M–H]^–^	−0.06	**C**_**24**_ **H**_**44**_ **O**_**10**_	294, 193, **173**	4-Feruloyl-quinic alkyl ester	L	^13^
**Flavonoids**									
**23**	5.33	449.1092	[M–H]^–^	−0.59	**C**_**21**_ **H**_**22**_ **O**_**11**_	287, **151**, 135	Eriodictyol hexoside	S, F	^40^
**24**	5.49	449.1095	[M–H]^–^	−1.26	**C**_**21**_ **H**_**22**_ **O**_**11**_	287, **151**, 135	Eriodictyol hexoside	S, F	^40^
**25**	6.08	481.0981	[M + H]^+^	−0.9	**C**_**21**_ **H**_**20**_ **O**_**13**_	**319**, 273, 243, 217	Myricetin hexoside	S, F	^41^
**26**	6.57	447.0931	[M–H]^–^	0.41	**C**_**21**_ **H**_**20**_ **O**_**11**_	**285**, 151, 135	Luteolin hexoside	S, F	^42^
**27**	7.13	493.0995	[M–H]^–^	−1.49	**C**_**22**_ **H**_**22**_ **O**_**13**_	**331**, 316, 287	Myricetin hexoside methyl ether	S, F, L	
**28**	7.01	451.1239	[M + H]^+^	−0.92	**C**_**21**_ **H**_**22**_ **O**_**11**_	**289**, 163, 153	Eriodictyol hexoside	S, F	Mass Bank
	7.60	449.1105	[M–H]^–^	−3.48	**C**_**21**_ **H**_**22**_ **O**_**11**_	**287**, 151, 135		S, F	^40^
**29**	7.66	447.0972	[M–H]^–^	−2.31	**C**_**21**_ **H**_**20**_ **O**_**11**_	284, 285, **255**, 256, 227	Kaempferol 3-hexoside	S, L	^36^
**30**	7.82	479.1187	[M + H]^+^	−0.62	**C**_**22**_ **H**_**22**_ **O**_**12**_	**317**, 302, 85	Isorhamnetin hexoside	S, L	^43^
**31**	7.00	433.1135	[M–H]^–^	1.2	**C**_**21**_ **H**_**22**_ **O**_**10**_	271, **135**	3’,4’,7-Trihydroxy-flavanone hexoside		HMDB
	7.90	435.1304	[M + H]^+^	−4.21	**C**_**21**_ **H**_**22**_ **O**_**10**_	**273**, 137, 163		S, F	HMDB
**32**	8.82	475.1264	[M–H]^–^	−3.81	**C**_**23**_ **H**_**24**_ **O**_**11**_	271, **135**, 91	3’,4’,7-Trihydroxy-flavanone hexoside acetate	S, F	
**33**	9.21	289.0709	[M + H]^+^	−0.82	**C**_**15**_ **H**_**12**_ **O**_**6**_	**289**, 163, 153	Eriodictyol		^43^
	9.17	287.0572	[M–H]^–^	−3.78	**C**_**15**_ **H**_**12**_ **O**_**6**_	287, 151, **135**, 95		S, F	^44^
**34**	9.63	285.0419	[M–H]^–^	−1.17	**C**_**12**_ **H**_**10**_ **O**_**6**_	199, 175, **133**, 107	Luteolin	S, F, L	^18^
**35**	9.76	611.1429	[M–H]^–^	−3.71	**C**_**30**_ **H**_**28**_ **O**_**14**_	323, **287**, 179, 177, 161, 151, 135	Eriodictyol caffeoyl hexoside	S, F	^36^
**36**	10.03	595.1455	[M–H]^–^	0.36	**C**_**30**_ **H**_**28**_ **O**_**13**_	323, **271**, 179, 161, 135	3’,4’,7-Trihydroxy-flavanone caffeoyl hexoside	F	
**37**	10.53	271.0622	[M–H]^–^	−3.69	**C**_**15**_ **H**_**12**_ **O**_**5**_	**135**, 91	3’,4’,7-Trihydroxy-flavanone	S, F	PubChem
**38**	12.43	301.0718	[M + H]^+^	−3.78	**C**_**16**_ **H**_**12**_ **O**_**6**_	**301**, 286, 258, 124	Trihydroxy-methoxy-flavone	F, L	^44^
	13.35	299.0559	[M–H]^–^	0.71	**C**_**16**_ **H**_**12**_ **O**_**6**_	299, **284**, 256			^44^
**39**	13.16	283.0616	[M–H]^–^	−1.4	**C**_**16**_ **H**_**12**_ **O**_**5**_	**268**, 240, 211, 117, 65	Dihydroxy-methoxy-isoflavone	L	^45^
**Other aromatic compounds**									
**40**	4.51	349.0612	[M–H]^–^	−3.78	**C**_**13**_ **H**_**18**_ **O**_**9**_ **S**	241, **97**, 83, 80, 71	Benzyl sulfohexoside	F, L	^15^
**41**	8.83	549.2360	[M–H]^–^	−3.39	**C**_**28**_ **H**_**38**_ **O**_**11**_	**549**, 161, 133, 71	Medioresinol hexoside	L	^16^
**42**	8.97	95.0493	[M + H]^+^	−1.69	**C**_**6**_ **H**_**6**_ **O**	**67**, 65	Phenol	F, L	^46^
**Terpenoids**									
**43**	5.69	305.1072	[M–H]^–^	−2.1	**C**_**13**_ **H**_**22**_ **O**_**6**_ **S**	305, **97**	Sulfotrihydroxy-megastigmadiene	S, L	^11^
**44**	6.02	433.2095	[M + FA–H]^–^	−3.64	**C**_**19**_ **H**_**32**_ **O**_**8**_	**433**, 387, 225, 207, 179, 161	Megastigman-3,9-dione hexoside	S, L	^47^
**45**	10.00	543.2810	[M + FA–H]^–^	0.16	**C**_**26**_ **H**_**42**_ **O**_**9**_	497, **335**	16,17-Dihydroxy-KA hexoside	F, L	^12^
**46**	10.98	428.2285	[M+NH_4_]^+^	−1.42	**C**_**21**_ **H**_**30**_ **O**_**8**_	411, 263, **245**, 227, 199	1-Acetoxy-4,9-dihydroxy-6-isobutyroxy-eudesmanolide	F	^10^
**47**	12.53	419.2069	[M + H]^+^	−1.12	**C**_**23**_ **H**_**30**_ **O**_**7**_	359, 263, 245, **227**, 199, 181	Unidentified eudesmanolide	S, F, L	
**48**	12.61	793.4412	[M–H]^–^	−4.05	**C**_**42**_ **H**_**66**_ **O**_**14**_	**631**, 613, 569	Protopanaxatriol hexoside	S, F	^48^
**49**	12.68	285.2220	[M + H]^+^	−2.49	**C**_**20**_ **H**_**28**_ **O**	267, 257, 229, 105, **83**, 69, 55	Retinal	S, F	^49^
**50**	12.99	470.2376	[M+NH4]^+^	1.83	**C**_**23**_ **H**_**32**_ **O**_**9**_	411, 245, **227**, 199, 181	Trilobolide-6-isobutyrate	F, L	^10^
**51**	13.21	445.2221	[M + H]^+^	−0.05	**C**_**25**_ **H**_**32**_ **O**_**7**_	245, **227**, 199, 181	Unidentified eudesmanolide	L	
**52**	13.35	447.2031	[M + H–H_2_O]^+^	−3.93	**C**_**24**_ **H**_**32**_ **O**_**9**_	405, 287, 245, **227**, 199, 83	Diacetoxy-tigloyloxy-eudesmanolide	F, L	^10^
**53**	13.42	433.2236	[M + H–H_2_O]^+^	−3.52	**C**_**24**_ **H**_**34**_ **O**_**8**_	263, **245**, 227, 199, 83	6-Isobutyroxy-1,4-dihydroxy-8-angeloyloxy-eudesmanolide	F, L	^10^
**54**	13.47	447.2378	[M + H]^+^	−0.16	**C**_**25**_ **H**_**34**_ **O**_**7**_	401, **245**, 227, 199, 184, 169	Unidentified eudesmanolide	L	
**55**	13.53	470.2755	[M+NH_4_]^+^	−1.4	**C**_**24**_ **H**_**36**_ **O**_**8**_	435, **245**, 227, 199, 181	Unidentified eudesmanolide	F, L	
**56**	13.88	496.2551	[M+NH_4_]^+^	−2	**C**_**25**_ **H**_**34**_ **O**_**9**_	461, 245, **227**,** 181**, 69	Acetoxy-methacryloxy-isobutyroxy-hydroxy-eudesmanolide	F	^10^
**57**	14.08	393.1914	[M + H]^+^	−1.58	**C**_**21**_ **H**_**28**_ **O**_**7**_	263, 245, **227**, 199	Acetyl-isobutyroxy-germacrenolide	F, L	^50^
**58**	14.16	475.2331	[M + H]^+^	−0.96	**C**_**26**_ **H**_**34**_ **O**_**8**_	415, 263, 245, 227, 181, **83**, 55	1-Acetoxy-4-hydroxy-6-isobutyryloxy-9-angeloyloxy-eudesmanolide	S, F, L	^10^
**59**	14.26	512.2841	[M+NH_4_]^+^	2.56	**C**_**26**_ **H**_**38**_ **O**_**9**_	495, 477, 245, **227**,** 181**, 85	1-Acetoxy-4-hydroxy-6-isobutyryloxy-9-isovaleryloxy-eudesmanolide	F	^10^
**60**	14.27	287.2374	[M + H]^+^	−1.6	**C**_**20**_ **H**_**30**_ **O**	269, 213, 105, 95, **81**, 69, 55	Retinol	F, L	^49^
**61**	14.31	359.2206	[M + H]^+^	3.03	**C**_**22**_ **H**_**30**_ **O**_**4**_	**299**, 281, 189, 149, 83, 69	1-Acetoxy-kaur-11(12),16dien-9-oic acid	S, L	^51^
**62**	14.45	415.2506	[M–H]^–^	−3.58	**C**_**25**_ **H**_**36**_ **O**_**5**_	415, **99**	3-Angloyloxy-hydroxy-KA	S, F, L	^12^
**63**	14.50	524.2859	[M+NH_4_]^+^	−0.94	**C**_**27**_ **H**_**38**_ **O**_**9**_	507, 489, 245, 227, **181**, 83	Unidentified eudesmanolide	F, L	
**64**	14.69	463.2498	[M–H]^–^	−1.73	**C**_**29**_ **H**_**36**_ **O**_**5**_	463, **147**, 103	3-Cinnamoyl-17-hydroxy-KA (wedeldin A)	S, F, L	^12^
**65**	14.82	317.2126	[M–H]^–^	−1.2	**C**_**20**_ **H**_**30**_ **O**_**3**_	**317**, 299, 271	Hydroxy-KA	F, L	^12^
**66**	15.18	221.1907	[M + H]^+^	−3.22	**C**_**15**_ **H**_**24**_ **O**	203, 109, **95**, 81, 69, 55	Spathulenol	L	^2^
**67**	15.38	447.2560	[M–H]^–^	−4.28	**C**_**29**_ **H**_**36**_ **O**_**4**_	447, **147**, 103	3-Cinnamoyloxy-KA	F, L	^12^
**68**	15.53	299.2030	[M–H]^–^	−4.48	**C**_**20**_ **H**_**28**_ **O**_**2**_	**299**, 151	Grandiflorenic acid	S, F, L	^12^
**69**	15.87	399.2555	[M–H]^–^	−3.54	**C**_**25**_ **H**_**36**_ **O**_**4**_	399, **99**	3-Angeloyloxy-KA	F, L	^12^
**Carboxylic and fatty acids derivatives**									
**70**	0.93	191.0568	[M–H]^–^	−3.58	**C**_**7**_ **H**_**12**_ **O**_**6**_	**191**, 173, 127, 93	Quinic acid	F, L	^52^
**71**	8.72	467.2153	[M + FA–H]^–^	−4.06	**C**_**19**_ **H**_**34**_ **O**_**10**_	**421**, 289, 85, 113, 101, 59	1-Octen-3-yl primeveroside	S, L	^53^
**72**	9.06	395.1384	[M–H]^–^	2.52	**C**_**16**_ **H**_**30**_ **O**_**7**_ **P**_**2**_	**395**, 97, 80	3-Ethyl-7,11-dimethyldodeca-2,6,10-trienyl phosphono hydrogen phosphate	S, F, L	^54^
**73**	9.34	233.0853	[M–H]^–^	0.01	**C**_**10**_ **H**_**18**_ **O**_**4**_ **S**	**97**, 80	Decadienyl sulfate	S, L	PubChem
**74**	11.96	329.2346	[M–H]^–^	−3.79	**C**_**18**_ **H**_**34**_ **O**_**5**_	**329**, 229, 211, 171, 139	Trihydroxy-octadecenoic acid	S, F, L	^18^
**75**	12.10	181.1221	[M + H]^+^	1.14	**C**_**11**_ **H**_**16**_ **O**_**2**_	**181**, 163, 135, 107, 93,59	Jasmolone	L	^17^
**76**	14.57	279.2329	[M + H]^+^	−3.75	**C**_**18**_ **H**_**30**_ **O**_**2**_	173, 149, **95**, 81, 67	Octadecatrienoic acid	F	^55^
**77**	15.32	293.1790	[M–H]^–^	0.69	**C**_**14**_ **H**_**30**_ **O**_**4**_ **S**	**97**, 80	Myristyl sulfate	F, L	PubChem
**Lipids**									
**78**	13.39	316.2839	[M + H]^+^	2.29	**C**_**18**_ **H**_**37**_ **NO**_**3**_	**316**, 298, 280, 60	Trihydroxy-sphingenine	F	^56^
**79**	13.82	721.3668	[M–H]^–^	−2.2	**C**_**33**_ **H**_**56**_ **O**_**14**_	**721**, 675, 415, 397, 277	Dihexosyl monoacyl glycerol (18:3)	S, L	^36^
**80**	14.40	476.2802	[M–H]^–^	−4.06	**C**_**23**_ **H**_**44**_ **NO**_**7**_ **P**	476, 279, 171, 153, **79**	Monoacyl phosphatidylethanolamine (18:2)	F	^18^
**81**	14.57	520.3387	[M + H]^+^	2.05	**C**_**26**_ **H**_**50**_ **NO**_**7**_ **P**	**520**, 502, 184, 104, 86	Monoacyl phosphatidyl choline glycerol (18:2)	F, L	^57^
**82**	14.60	579.2861	[M–H]^–^	−2.83	**C**_**27**_ **H**_**48**_ **O**_**11**_ **S**	**279**, 225, 165, 153, 81	Sulfoquinovosyl monoacyl glycerol (18:2)	L	^18^
**83**	14.76	571.2886	[M–H]^–^	0.5	**C**_**25**_ **H**_**49**_ **O**_**12**_ **P**	**571**, 255, 241, 153, 79	Monoacyl phosphatidylinositol (16:0)	F, L	^18^
**84**	14.86	555.2876	[M–H]^–^	−5.26	**C**_**25**_ **H**_**48**_ **O**_**11**_ **S**	255, 225, **81**	Sulfoquinovosyl monoacyl glycerol (16:0)	S, L	^18^
**85**	15.03	507.2722	[M–H]^–^	1.27	**C**_**24**_ **H**_**45**_ **O**_**9**_ **P**	**279**, 153, 79	Monoacyl phosphatidyl glycerol (18:2)	L	^18^
**86**	15.90	793.5130	[M–H]^–^	1.41	**C**_**41**_ **H**_**78**_ **O**_**12**_ **S**	**793**, 317, 225, 165, 81	Sulfoquinovosyl diacyl glycerol (16:0/16:0)	L	^18^

**Fig. 3:**
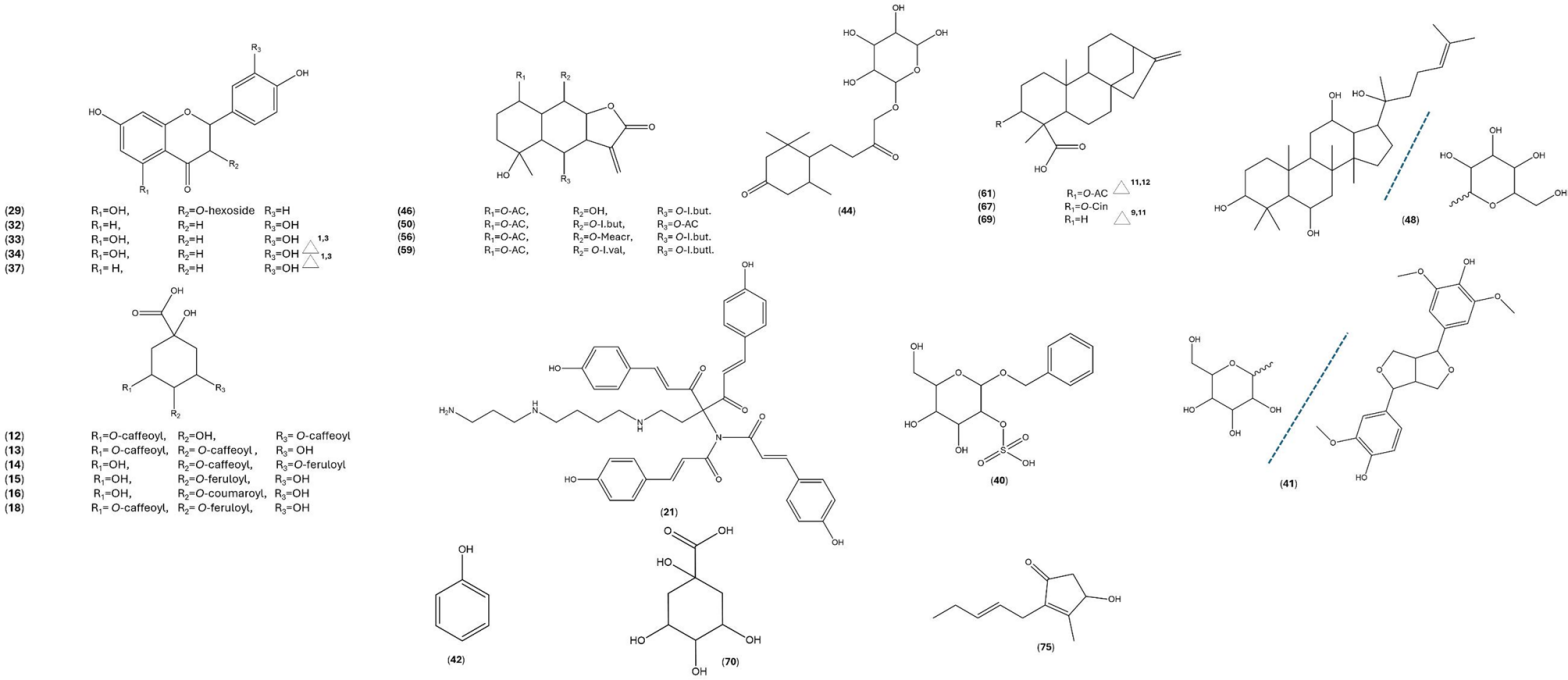
Structures of the putatively annotated metabolites in *S. trilobata* flowerheads and leaves and discussed throughout the study. Metabolites ID follows that listed in Table 1.

Among these, several compounds were reported for the first time in the genus, including phenolic acids **16**,** 17**,** 20**,** & 21**; flavonoids **23**,** 31**,** 32**, **33**, **35**, **36**, & **37**; and terpenoids **43**, **48**, **49**, **57**, & **60**. Additionally, compounds **40** and **56** were detected in *S. trilobata* for the first time. The chemical structures of the metabolites discussed throughout this study are displayed in Fig. 3.The detection was mode-specific; terpenoids were clearly detected in the positive ionization mode, while flavonoids and phenolic acids were distinctly recognized in the negative mode.

#### Flavonoids

Flavonoids are a class of secondary metabolites that are widely distributed in different organs of the plant, especially flowers, and are responsible for many functions such as regulating cell growth and attracting pollinators^5^. They were efficiently annotated in both negative and positive modes, however, negative ion spectra have offered improved sensitivity for the analysis of flavonoids.

Thirteen flavonoids were detected in negative mode, while four flavonoids were observed in positive mode. The putatively annotated flavonoids are subclassified into nine flavanones, four flavonols, three flavones, and one isoflavone. It was observed that flavanones represent the major percentage of the identified flavonoids.

The nomenclature of product ions that yielded from the fragmentation of the flavonoids suggested by Ma et al. (1997 and 1999) is applied. ^m, n^A and ^m, n^B labels are utilized to represent the product ions that contain intact A and B rings, respectively, while the superscript m and n express the C-ring bonds that have been broken by retro-Diels-Alder reactions^6,7^ as shown in **Fig.** (**S1**).

In detail, the flavanone structures were confirmed, in the negative mode, based on retro-Diels-Alder cleavage that resulted in two major fragment ions, ^1,3^A^–^ and ^1,3^B^–^ at *m/z* 151 and 135, respectively, characteristic for eriodictyol (**33**) [*m/z* 287.0572 (C_15_H_11_O_6_)^–^] (**Fig. S2**). Also, ^1,3^B^–^ and ^0,4^B^–^ at *m/z* 135 and 91 were diagnostic for 3’,4’,7-trihydroxy-flavanone (**37**) [*m/z* 271.0622 (C_15_H_11_O_5_)^–^] (**Fig. S3**).

On the other side, the mass spectrum of luteolin (**34**) [*m/z* 285.0419 (C_12_H_9_O_6_)^–^] demonstrated two diagnostic peaks at ^1,3^A^–^ and ^1,3^B^–^ at *m/z* 151 and 133, respectively, in addition to the appearance of other peaks at *m/z* 175 and 199, corresponding to [M–H–C_3_O_2_–C_2_H_2_O]^–^ and [M–H–C_2_H_2_O–CO_2_]^–^, respectively (**Fig. S4**). It is noteworthy that flavonoids are vastly found in their glycosidic forms, where they are bound to one or more sugar moieties.

When the flavonoid glycosides fragmented in negative ion mode, the detachment of the sugar unit typically generates the most abundant aglycone ion [Ag]^–^. The mass of this aglycone serves as a key identifier for the specific flavonoid, such as *m/z* 287 (eriodictyol), 273 (3’,4’,7-trihydroxy-flavanone), 285 (luteolin), and 319 (myricetin). Notably, the MS^2^ spectrum of kaempferol 3-hexoside (**29**) [*m/z* 447.0972 (C_21_H_19_O_11_)^–^] demonstrated a more abundant fragment at *m/z* 284, corresponding to [Ag–H]^–^, compared to the aglycone ion [Ag]^–^ at *m/z* 285. Moreover, the presence of the [Ag–H–CO–H]^–^ ion at *m/z* 255 further supports 3-glycosylation (**Fig. S5**). In addition to these putatively annotated compounds, other flavonoid glycoside forms were observed.

Compound **32** [*m/z* 475.1264 (C_21_H_21_O_10_)^–^] exhibited a highly intense fragment, [Ag]^–^, at *m/z* 271 **(**3’,4’,7-trihydroxy-flavanone) after the loss of [162 + 42], suggesting the presence of an acetyl group attached to the sugar moiety. This led to its annotation as 3’,4’,7-trihydroxy-flavanone hexoside acetate (**Fig. S6**).

Additionally, flavonoid glycosides featuring conjugations between sugar and cinnamoyl groups such as compound **35** [*m/z* 611.1429 (C_30_H_27_O_14_)^–^] revealed intense peaks at *m/z* 287, 323, 179, and 135, which affirmed its identification as eriodictyol caffeoyl hexoside (**Fig. S7**). Similarly, the fragmentation pattern of compound **36** [*m/z* 595.1455 (C_30_H_27_O_13_)^–^] exhibited highly dominant peaks consistent with those of compound **35**, except for the aglycone peak at *m/z* 271, identifying it as trihydroxy-flavanone caffeoyl hexoside Table 1. Notably, these acyl flavonoids are reported for the first time in the plant, while myricetin, luteolin, and kaempferol have been previously detected within the *Sphagneticola* genus^8^.

#### Terpenoids

Terpenoids are considered the most abundant secondary metabolites in the plant. They are involved in different interactions and defense mechanisms between plants and microorganisms^9^. Our study revealed that terpenoids constitute the largest proportion of the identified compounds, with a total of twenty-seven terpenoids.

These detected terpenoids were divided into three groups: sixteen sesquiterpenoids, ten diterpenoids, and one triterpenoid. The positive ionization mode proved particularly effective for the analysis of terpenoids, especially sesquiterpenoids. Diterpenoids, on the other hand, were detected in both negative and positive modes, each exhibiting unique fragmentation patterns.

##### Sesquiterpenoids

A total of sixteen sesquiterpenoids, predominantly from the eudesmanolide class, were detected in *S. trilobata* and were preferentially ionized under positive ionization conditions.

The discussion provides an illustrative image of the fragmentation processes of eudesmanolides with bulky substituents that are mainly attached to C-1, C-6, and C-9, leading to the formation of unstable molecular ions. The compositions of the fragment ions have been identified, and it has been observed that the breakdown of the lactone skeleton occurs only after the removal of these bulky substituents. The scheme in **Fig.** (**S8**), adapted from Dias et al. (2017), outlines the sequential fragmentation of eudesmanolides following the loss of these voluminous substituents^10^.

For instance, Compound **50** [*m/z* 470.2376 (C_23_H_33_O_9_)^+^] as an ammonium adduct], which displayed high abundance in *S. trilobata* extracts, demonstrated a distinct peak at *m/z* 263 after loss of two acetyl, one isobutyl, and two water molecules [M–2*42–70–2*18]^+^. Additionally, two diagnostic peaks at *m/z* 245 and 227 were observed, characteristic of eudesmanolides with exocyclic methylene attached to C-11 after the neutral loss of water (**Fig. S9**). Trilobolide-6-isobutyrate (**50**) is considered one of the major metabolites previously reported in *S. trilobata*.

Similar fragmentation patterns, with prominent peaks at *m/z* 263, 245, and 227, were observed in *m/z* in the mass spectra of compounds **46** [*m/z* 428.2285 (C_21_H_30_O_8_)], **56** [*m/z* 496.2551 (C_25_H_34_O_9_)], and **59** [*m/z* 512.2841 (C_26_H_38_O_9_)], all detected as ammonium adducts. These peaks were observed after the loss of specific substituents: acetyl and isobutyl groups for compound **46**, acetyl, isobutyl, and methacryl for compound **56**, and acetyl, isobutyl, and isovaleryl moieties for **59** (**Fig. S10**). Except for compound **56**, which has only been reported within the genus, all eudemasnolides annotated in this study have been previously isolated from the plant^2^.

Among other types of annotated sesquiterpenoids, two megastigmene compounds were annotated in the negative mode. Compound **43** [*m/z* 305.1072 (C_13_H_22_O_6_S)] displayed two characteristic peaks at *m/z* 97 and 80, corresponding to the sulfate and sulfonate ions, respectively, and was putatively annotated as sulfotrihydroxy-megastigmadiene. Its fragmentation manner concurred with the reported data^11^. Additionally, the mass spectrum of compound **44** [*m/z* 433.2095 (C_19_H_32_O_8_) as a formic acid adduct] displayed a highly intense ion at *m/z* 225 after the loss of a sugar moiety, and a peak at *m/z* 207, corresponding to [M–H–162–18]^–^ (**Fig. S11**). This compound was assigned as megastigman-3,9-dione hexoside.

##### Diterpenoids

Conversely, a total of ten diterpenoids, all based on the KA skeleton, were more effectively observed in the negative mode, compared to the positive mode. Among these, three compounds were detected under positive ionization, while the remaining seven were recognized under negative ionization.

Our analysis revealed that KA derivatives exhibit a distinct fragmentation pattern in the negative mode, characterized primarily by the loss of substituents such as cinnamoyl, angeloyl, or sugar moieties. Notably, KA derivatives did not undergo fragmentation of the parent ion (KA) in the negative mode, which may serve as a unique diagnostic feature for their detection^12^.

In detail, the mass spectrum of compound **68** [*m/z* 299.2030 (C_20_H_27_O_2_)^–^] displayed mainly the molecular ion peak at *m/z* 299 (**Fig. S12**) and was recognized as grandiflorenic acid, one of the major reported diterpenoids in the plant. Likewise, compound **65** [*m/z* 317.2125 (C_20_H_29_O_3_)^–^] revealed a small fragment ion at *m/z* 299 after the loss of water and was putatively annotated as hydroxy-KA Table 1. The detected KA derivatives showed considerable structural diversity in their attached groups, which vary from hydroxy groups to bulky side chains.

For instance, KA derivatives with bulkier substituents at C-3, compound **69** [*m/z* 399.2555 (C_25_H_35_O_4_)^–^] unveiled a minor peak at *m/z* 317 [M–H–83]^–^ due to the loss of angeloyl side chain, along with an abundant peak at *m/z* 99, representing the angeloyloxy substituent (**Fig. S13**). This compound was putatively annotated as 3-angeloyloxy-KA. The diterpene **67** [*m/z* 447.2560 (C_29_H_35_O_4_)^–^] showed two intense peaks at *m/z* 147 and 103, corresponding to the cinnamoyl moiety and its decarboxylated ion, respectively, suggesting its identity as 3-cinnamoyloxy-KA (**Fig. S14**).

Conversely, in positive ionization mode, KA derivatives displayed a more complex fragmentation pattern, generating a variety of fragment ions. Compound **61** [*m/z* 359.2206 (C_22_H_31_O_4_)^+^] exhibited a more complex fragmentation pattern with multiple fragment ions in the positive mode. For instance, the peak at *m/z* 299 corresponded to [M + H–42–18]^+^ ion due to the losses of acetyl and water moieties. Additional peaks at *m/z* 281, 253 were observed, formed through the sequential loss of carbonyl and water molecules, respectively, (**Fig. S15**). The compound was annotated as 1-acetoxy-kaur-11(12),16dien-9-oic acid, marking its first reported detection in the plant.

##### Triterpenoids

One triterpene (**48**) was detected in the negative mode [*m/z* 793.4412 (C_42_H_65_O_14_)^–^], showing three ion peaks at *m/z* 631, 613, and 569 due to the sequential losses of a hexose moiety [M–H–162]^–^, water molecule [M–H–18]^–^, and carboxylate group [M–H–44]^–^, respectively, (**Fig. S16**). This compound was putatively annotated as protopanaxatriol hexoside. It is worth noting that it is the first detection of this triterpenoid in the plant.

#### Phenolic acids

A total of thirteen phenolic acids were observed in *S. trilobata*, namely, twelve compounds in the negative ionization and one in the positive one. UPLC-MS analysis further revealed a notable abundance of chlorogenic acids within the plant’s extracts.

In total, ten chlorogenic acid derivatives were detected. Among these were two dicaffeoyl-quinic acid isomers, compounds **12** [*m/z* 515.1220 (C_25_H_23_O_12_)^–^] and **13** [*m/z* 515.1219 (C_25_H_23_O_12_)^–^]. The base peak at *m/z* 191 confirmed the assignment of compound **12** as 3,5-dicaffeoyl-quinic acid (**Fig. S17**), while the characteristic base peak at *m/z* 173 is indicative of compound **13**, assigned as 4,5-dicaffeoyl-quinic acid (**Fig. S18**), consistent with the literature^13^. Both isomers were previously isolated from *S. trilobata*^2^.

Moreover, the two caffeoyl-feruloyl-quinic acid isomers **14** and **18** [*m/z* 529.1370 (C_26_H_25_O_12_)^–^] were differentiated by their base peak at *m/z* 173 and the relative abundance of their fragment ion at m/z 335, consistent with established fragmentation patterns^13^. For isomer **14**, the intensity of the *m/z* 335 fragment was not less than 40% of the base peak, supporting its identification as 3-feruloyl-4-caffeoyl-quinic acid (**Fig. S19**). In contrast, the *m/z* 335 fragment for isomer **18** did not exceed 20% of the base peak, leading to its assignment as 4-feruloyl-5-caffeoyl-quinic acid (**Fig. S20**).

Two additional chlorogenic acids, compounds **15** and **16**, were putatively annotated as 4-coumaroyl-quinic acid [*m/z* 337.0941 (C_16_H_17_O_8_)^–^] and 4-feruloyl-quinic acid [*m/z* 367.1048 (C_17_H_19_O_9_)^–^], respectively (**Fig. S21**,** S22**). The presence of a base peak at *m/z* 173 in both, corresponding to dehydrated quinic acid, confirmed the substitution at the C-4 position^13^.

In the positive ionization mode, one phenolic compound was detected and annotated as tetra-coumaroyl spermine **21** [*m/z* 787.3677(C_46_H_51_N_4_O_8_)^+^]. The mass spectrum revealed key peaks at *m/z* 641, 495, and 204, corresponding to the sequential loss of coumaroyl moieties^14^(**Fig. S23**).

#### Other aromatic compounds

Three aromatic compounds were observed, with two detected in negative ionization mode and one in positive ionization mode. Compound **40** [*m/z* 349.0612 (C_13_H_17_O_9_S)^–^] exhibited high peaks at *m/z* 97 and 80, corresponding to sulfate and sulfonate ions, respectively. Also, another peak was observed at *m/z* 241, indicative of a hexose moiety attached to the sulfate group (**Fig. S24**). Accordingly, the compound was recognized as benzyl sulfohexoside, previously isolated from the genus^15^.

Moreover, compound **41** [*m/z* 549.2360 (C_28_H_37_O_11_)^–^] revealed a base peak at *m/z* 161 (a hexose moiety), and was annotated as medioresinol hexoside (**Fig. S25**) and its identification was consistent with the reported literature^16^.

#### Carboxylic and fatty acids derivatives

Regarding carboxylic acids, quinic acid **70** [*m/z* 191.0568 (C_7_H_12_O_6_)^–^] showed characteristic peaks at *m/z* 191, 173, 127, and 85 (**Fig. S26**). MS^2^ spectrum of jasmolone **75** [*m/z* 181.1221(C_11_H_17_O_2_)^+^] revealed several peaks including *m/z* 163 and 135 due to the gradual neutral losses of water and carbonyl group^17^(**Fig. S27**).

Lipophilic peaks appeared in the late elution region of the UPLC/MS chromatogram, including fatty acyl and fatty acids derivatives. For instance, hydroxylated fatty acid was determined as trihydroxy-octadecenoic acid **74** [*m/z* 329.2346 (C_18_H_33_O_5_)^–^] showing several losses of water molecules^18^(**Fig. S28**).

#### Lipids

Lipids are widely found in the plant kingdom, playing a crucial role in growth and the structural composition of cell membranes^19^. In this study, nine lipids were recorded, mostly in the negative mode, and categorized into four phospholipids, three sulfolipids, one sphingolipid and one glycerolipid. Considering phospholipids, some specific groups such as choline, myoinositol, or ethanolamine, may be attached to the phosphate, leading to a wide variation of phospholipid structures.

In the negative mode, compound **80** [*m/z* 476.2802 (C_23_H_43_NO_7_P)^–^] showed crucial peaks at *m/z* 279 (octadecadienoic acid (18:2)), 196 (phosphatidyl-ethanolamine), 153 (phospho-glycerol), and 79 (phosphate). Therefore, it was assigned as monoacyl phosphatidyl-ethanolamine (18:2) (**Fig. S29**). The mass spectrum of **83** [*m/z* 571.2885 (C_25_H_48_O_12_P)^–^] showed a base peak at *m/z* 255 (hexadecanoic acid (16:0)) and fragment ion at 241 (phosphatidyl-inositol), confirming its identification as monoacyl phosphatidylinositol (16:0).

Additionally, monoacyl phosphatidyl choline-glycerol (18:2) (**81**) [*m/z* 520.3387 (C_26_H_51_NO_7_P)^**+**^] was also annotated based on the presence of diagnostic peaks at *m/z* 184 (phosphatidyl-choline) and 104 (choline) (**Fig. S30**). On the other hand, *m/z* 225 (sulfoquinovosyl–18) and 81 (sulfate ion) were characteristic peaks for sulfolipids which can be categorized as sulfoquinovosyl lipids where the sulfate sugar group (quinovose) is attached to the acyl glycerol.

Three sulfoquinovosyl glycerol were assigned, namely, **82** [*m/z* 579.2861 (C_27_H_47_O_11_S)^–^], **84** [*m/z* 555.2876 (C_25_H_47_O_11_S)^–^], and **86** [*m/z* 793.5130 (C_41_H_77_O_12_S)^–^] by identifying characteristic peaks at *m/z* 225 and 81. Also, Compounds **82**, **84**, and **86** were assigned as sulfoquinovosyl monoacyl glycerol (18:2), sulfoquinovosyl monoacyl glycerol (16:0), and sulfoquinovosyl diacyl glycerol (16:0/16:0), respectively, (**Fig. S31**).

### Multivariate data analyses of UPLC-ESI-QTOF-MS/MS dataset

Having catalogued the individual phytochemical constituents, we next employed multivariate statistical analysis to reduce dimensionality of the untargeted dataset (> 1,600 features), systematically differentiate the organ-specific metabolic profiles, and identify the key compounds driving this chemical divergenc.

Principal component analysis (PCA) provided an unsupervised overview of sample clustering without prior knowledge of the dataset, orthogonal partial least-squares discriminant analysis (OPLS-DA) highlighted organ-discriminating features and further validated the PCA results, and partial least-squares analysis (PLS) enabled direct correlation of metabolite features with antioxidant assays.

**Fig. 4:**
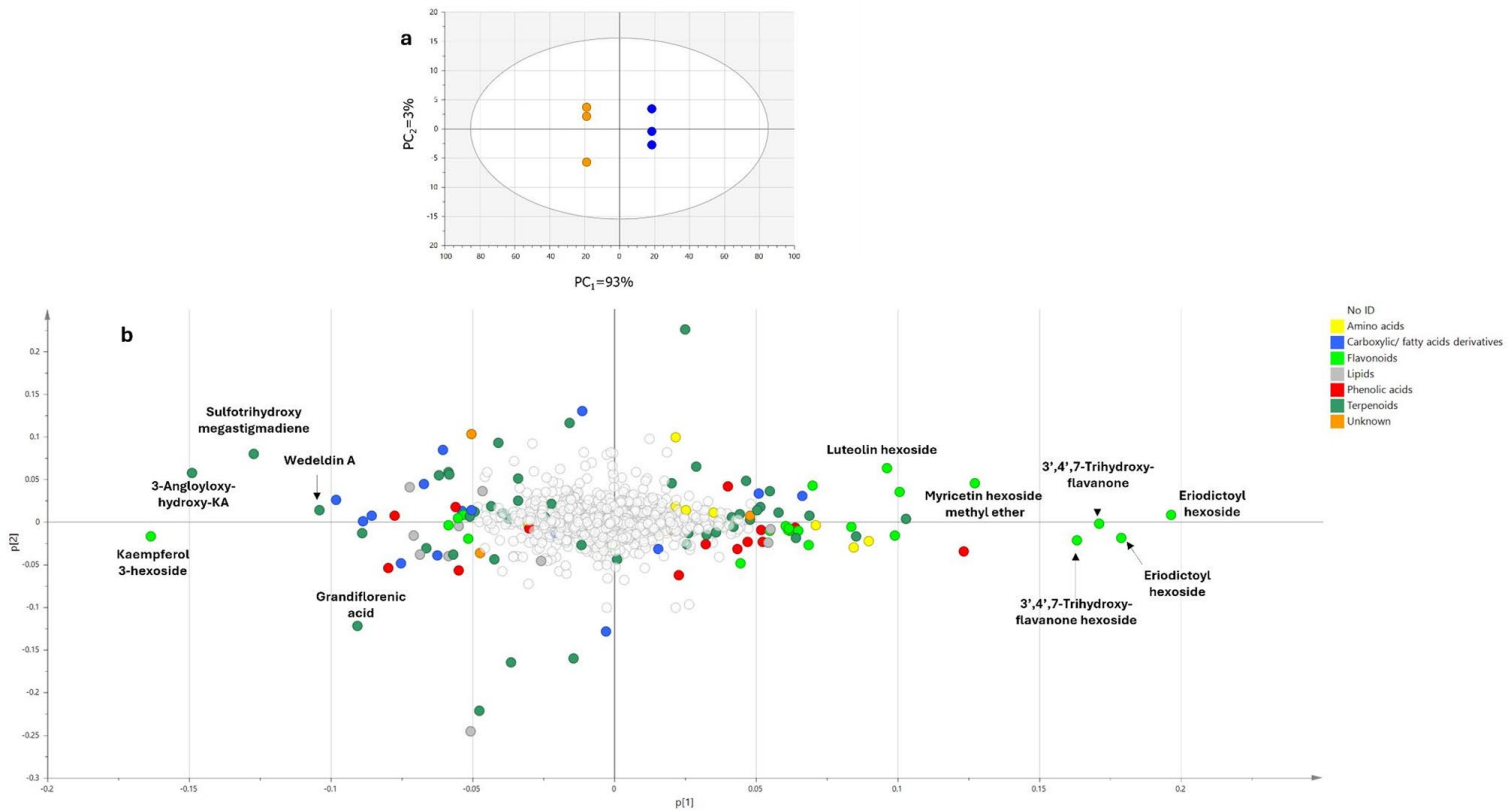
UPLC-MS based principal component analysis (PCA) of ethanol extracts from *S. trilobata* leaves ($\color{red} {\bullet}$) and flowerheads ($\color{blue} {\bullet}$) (*n* = 3) (**a)**. Score Plot of PC1 vs. PC2. (**b)**. Loading plot for PC1 and PC2.

To determine the relative phytochemical profile variance between *S. trilobata* flowerheads and leaves, the LC-MS dataset of 6 columns (2 organs with 3 replicates) × 1666 rows (peak abundance of the statistically significant compounds compared to blank) was investigated by PCA on the complete run time (R_t_ 0–17 min). The main principal component (PC) to differentiate between samples (PC1) accounts for 93% and PC2 for 3%. Our findings revealed that flowerheads and leaves constitute two different metabolic profiles Fig. 4.

Thoroughly, the score plot Fig. 4a shows that the leaf replicates are present on the left side or the negative PC1 values indicating their adequate separation from the replicates of flowerheads that appeared on the right side (positive PC1 values). The loading plot Fig. 4b further highlighted specific compounds contributing to this discrimination. Flavonoids such as eriodictyol hexoside (**28** and **24**), 3’,4’,7-trihydroxy-flavanone and its glycoside (**31** and **37**), myricetin hexoside methyl ether (**27**), and luteolin a hexoside (**34**) were predominantly detected on the right side, indicating their association with the flowerhead.

In contrast, terpenoids such as 3-angeloyloxy-hydroxy-KA (**62**), wedeldin A (**64**), and grandiflorenic acid (**68**) were observed on the left side, associated with the leaf. These findings underscore the significance of flavonoids and terpenoids as key classes of compounds for distinguishing between the metabolic profiles of the flowerheads and leaves. Additionally, OPLS-DA a supervised discriminant analysis technique, was also conducted to figure out the discriminant variables between the two organs and further validate the PCA results.

The S-plot (**Fig. S32**) illustrates the distinctive separation between flowerheads and leaves attributed to the enrichment of flowerheads with different flavonoids and the predominance of terpenoids in leaves. Markedly, kaempferol hexoside emerged as the only flavonoid putatively annotated as a discriminating metabolite in leaves.

### Antioxidant activity

Beyond establishing these distinct phytochemical signatures, it was essential to determine how they translate into functional bioactivity. Accordingly, we evaluated and compared the antioxidant capacity of the flowerheads and leaves to assess whether their divergent metabolite profiles correspond to measurable differences in biological activity.

The antioxidant capacity of *S. trilobata* flowerheads and leaves was evaluated using three complementary methods, the 2,2-diphenyl-1-picrylhydrazyl (DPPH), Ferric Reducing Antioxidant Power (FRAP), and 2,2′-azino-bis(3-ethylbenzothiazoline-6-sulfonic acid) (ABTS) assays (Table 2).

**Table 2:** Antioxidant activities of *S. trilobata* flowerheads and leaves. Results are expressed as means ± SD (*n* = 3). ^(a−c)^ Different letters in the same column donate significance (*p-*value < 0.05); DPPH; (2,2-diphenyl-1-picrylhydrazyl), FRAP; ferric reducing antioxidant power; ABTS, 2,2′-azino-bis(3-ethylbenzothiazoline-6-sulfonic acid.

Sample	DPPH IC_50_ (µg/mL)	FRAP µM Trolox Equivalent/mg extract	ABTS IC_50_ (µg/mL)
Flowerheads	427.00^a^ *±* 15.79	1000.67^a^ *±* 38.00	291.00^a^ *±* 6.80
Leaves	961.70^b^ *±* 8.95	313.67^b^ *±* 13.47	544.60^b^ *±* 45.50
Ascorbic acid	44.24^c^ *±* 0.91	-	50.97^c^ *±* 6.13

In the DPPH assay, the flowerheads exhibited a markedly stronger free radical scavenging ability, with an IC_50_ value of 427.00 µg/mL, which was more than two times lower than that of leaves (961.70 µg/mL). For context, the standard ascorbic acid had an IC_50_ of 44.24 µg/mL.

Regarding the FRAP assay, the antioxidant impact of the flowerheads surpassed the effect of leaves, with a reducing power of 1000.67 µM Trolox equivalent (TE)/mg, compared to 313.67 µM TE/mg for leaves.

This superior activity of flowerheads was further confirmed by ABTS assay, achieving IC_50_ values of 291.0 µg/mL and 544.6 µg/mL, respectively, compared to 50.97 µg/mL of ascorbic acid.

Although the results clearly demonstrated that flowerheads possess a significantly more potent antioxidant activity than leaves, these effects were relatively weaker compared to the standard. This is typical for crude extracts, which often demonstrate lower efficacy than pure reference compounds such as ascorbic acid; nevertheless, they remain promising as antioxidant supplements or sources for isolating more potent bioactive constituents.

### Correlation between metabolites and antioxidant activities

The marked disparity in antioxidant potential between the two organs strongly implies that specific phytochemicals are responsible for this enhanced activity. To pinpoint the exact metabolite drivers and elucidate the underlying structure**-**activity relationships, we performed an integrated PLS correlation analysis, linking the comprehensive metabolomic dataset (X variables) with the measured antioxidant responses (Y variables).

**Fig. 5:**
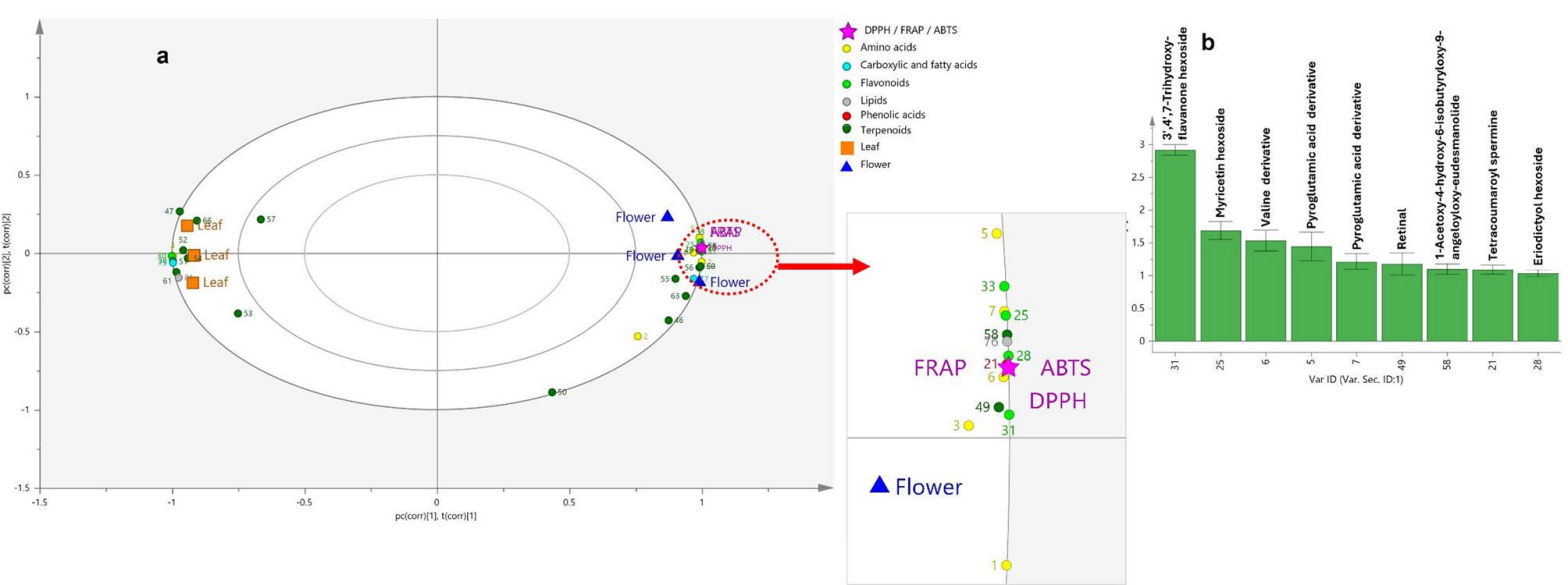
(a). UPLC-MS based PLS biplot represents the correlation between *S. trilobata* organs and antioxidant activity in positive mode. (**b)**. The putatively annotated metabolites in positive mode with variable importance to projections (VIP) values ≥ 1.

**Fig. 6:**
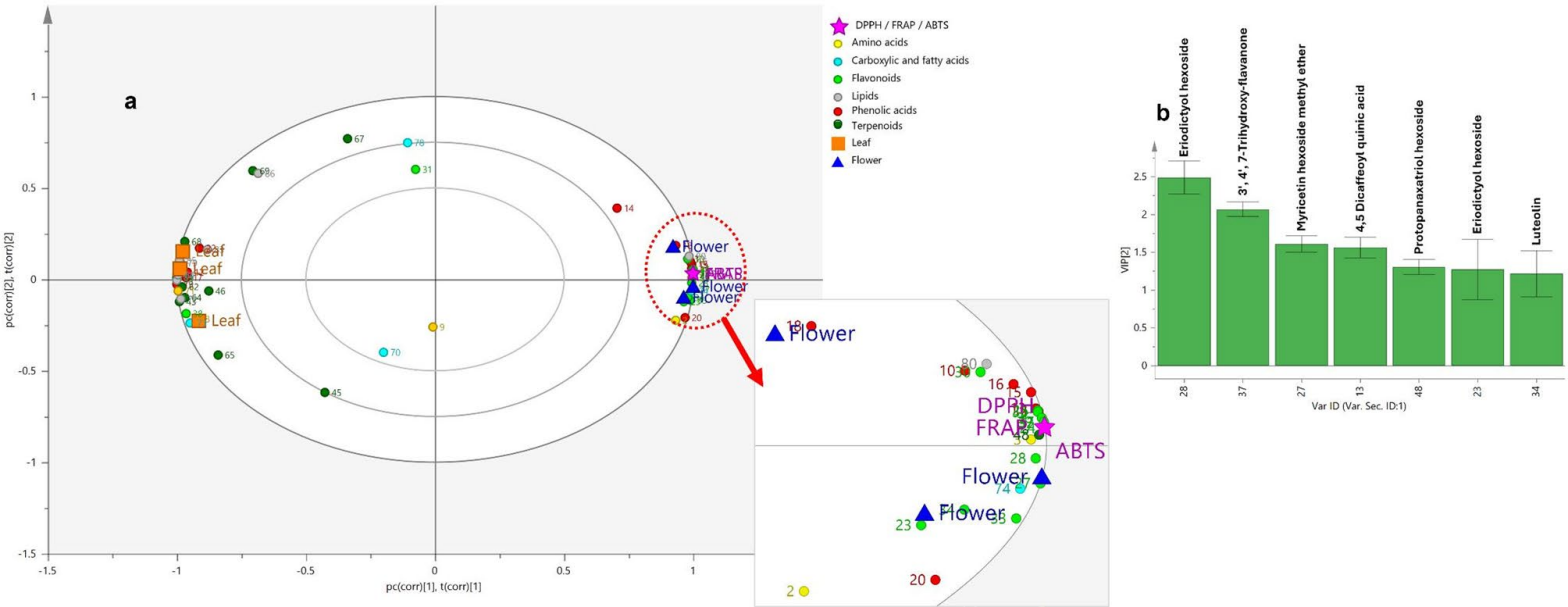
(a). UPLC-MS based PLS biplot represents the correlation between *S. trilobata* organs and antioxidant activity in negative mode. (**b)**. The putatively annotated metabolites in negative mode with variable importance to projections (VIP) values ≥ 1.

The resulting PLS biplot, integrating both score and loading plots in positive Fig. 5 and negative Fig. 6 modes, revealed a clear metabolic distinction between flowerheads and leaves, with the model demonstrating excellent fit and predictive validity (R^2^_Y_ > 99.8%, Q^2^ > 99.7%). Antioxidant activity (Y variables) was strongly associated with flowerheads, corroborating the in vitro findings Table 2. To pinpoint the metabolites driving this separation, we prioritized those with a variable importance in projection (VIP) score ≥ 1 Figs. 5b and 6b.

In the positive ionization mode, the key VIP metabolites included several flavonoids, specifically 3’,4’,7-trihydroxy-flavanone hexoside (**31**), myricetin hexoside (**25**), and eriodictyol hexoside (**28**), alongside pyroglutamic acid and valine derivatives (**5**, **6**, and **7**), retinal (**49**), and tetracoumaroyl sperimine (**21**).

On the other side, a related but distinct set of influential compounds emerged in the negative mode, featuring eriodictyol hexoside (**23**, **28**), 3’,4’,7-trihydroxy-flavanone (**37**), luteolin (**34**), myricetin hexoside methyl ether (**27**), and 4,5-dicaffeoyl-quinic acid (**13**). Together, these findings confirm that the most significant metabolites belong primarily to the classes of flavonoids, phenolic acids, and amino acids Figs. 5 and 6.

The potency of these putatively annotated metabolites is supported by their established structure–activity relationships. For instance, flavonoids like eriodictyol and myricetin possess a B-ring *ortho*-dihydroxy (catechol) group, which enhances radical stability and antioxidant power^20^.

Likewise, caffeoylquinic acids, with their catechol moiety, are more potent antioxidants than their feruloyl analogs^21^. The carotenoid retinal also plays a role, with its terminal aldehyde group associated with notable bioactivity^22^. Additionally, the antioxidant capacity of amino acids improves with increased lipophilicity, such as through esterification or the addition of methyl groups to the amine moiety^23^, explaining the prominence of such derivatives in the VIP list.

In summary, the integrated chemometric analyses demonstrate that the strong antioxidant activity observed in *S. trilobata* flowerheads is correlated with flavonoids, phenolic acids, and amino acids.

## Materials and methods

### Plant extraction

The flowerheads and leaves of *S. trilobata* were acquired from the Medicinal, Aromatic, and Poisonous Plants Experimental Station, Faculty of Pharmacy, Cairo University that has been registered in the New York Botanical Garden website (Herbarium code: CUFP). The plant was identified by Prof. Abd Elhalim Abd El-Mogali Mohamed, Professor of Plant Taxonomy and Flora, Agricultural Museum, Egypt, and a voucher specimen (3–10-2021I) was retained at the herbarium of the Department of Pharmacognosy, Faculty of Pharmacy, Cairo University. Sample collection was performed according to the institutional and national guidelines. No special permits were needed for the plant material collection.

The total ethanolic extracts were prepared individually from the air-dried powdered leaves (20 g) and flowers (20 g) by repeated maceration in 95% ethanol at room temperature (5 × 1 L, 2 days maceration time). After filtration, the ethanolic filtrates were collected and evaporated under reduced pressure to give 10 g of greenish-black dried residue for the leaf and 5 g for the flower. The difference in dry yields reflects genuine differences in extractable constituents between organs (leaves typically yield higher amounts of ethanol-soluble constituents than flowerheads). The total residue was kept in the refrigerator for further phytochemical and biological studies.

### Chemicals

Formic acid (≥ 95.0%), water, acetonitrile, and methanol were of LC-MS grade and procured from Sigma Aldrich (NSW, Australia). FRAP, DPPH, ABTS, Trolox, and ascorbic acid were supplied by Nawah Scientific (Cairo, Egypt). All other solvents were of analytical grade and provided by Piochem (Giza, Egypt).

### Sample preparation for UPLC-ESI-QTOF-MS/MS analysis

*S. trilobata* flowerheads and leaves extracts were dissolved in methanol (5 mg/mL) and filtered through a 0.2 μm PTFE filter for further analyses.

### UPLC-ESI-QTOF-MS/MS metabolite profiling

Analysis of the samples was conducted in both modes (positive and negative) using data-dependent “AutoMSMS” acquisition. The analysis was performed using an Agilent 1290 HPLC system interfaced with an Agilent 6546 quadrupole time-of-flight mass spectrometer with dual AJS electrospray ion source (ESI).

The applied parameters were as follows: narrow isolation width MS/MS of ~ 1.3 amu, 4 maximum precursors per cycle with dynamic exclusion activated after 2 spectra for 0.2 min. Intervals of MS and MS/MS (50–1200 m/z) were employed using constant collision energies set at 10, 20, and 40 eV. Gas and sheath gas temperatures of 320 °C °C and 350 °C, respectively, were applied, with the gas flow adjusted to 10 L/min and the sheath gas flow set to 35 L/min. The capillary voltage was 3500 V in positive mode and 4500 V in negative mode.

Moreover, other parameters, including the fragmentor, skimmer1, and octopoleRFPeak, were optimized to 120, 65, and 750, respectively, along with 1000 V nozzle voltage. Concurrent infusion of the Agilent TOF reference mass solution kit (G1969-85001) ensured accurate mass calibration.

The utilized flow rate was set at 0.28 mL/min. Elution was carried out with water (A) and methanol (B) as mobile phases, each containing 2% formic acid. The gradient elution was incorporated as follows; 0% B was applied for 1 m, elevated to 80% B at 16 min, set for 2 min, further dropped to 0% B at 18.1 min, followed by conditioning for 2 min. The utilized column was ACQUITY UPLC HSS-T3 (1.8 μm, 2.1 × 100 mm, Waters Corporation, Milford, USA) fitted with a 2.1 × 5 mm T3 VanGurdTM PreColumn (Waters Corporation, Milford, USA). 45 °C was the applied column temperature.

MSDIAL version 4.92 was adopted to process raw data. The negative and positive modes were handled individually, employing tolerances of MS1 and MS2 as follows: 0.01 and 0.025 Da, respectively, mass slice width of 0.1 Da, minimum peak height (1000 amplitude), and aligned to a pooled quality control reference (QC) with 0.05 min R_t_ tolerance and 0.015 Da MS1 tolerance^24^.

During MSDIAL processing for metabolite interpretation, version S17 of the Riken public spectral library was initially implemented. Features that were exported by MSDIAL were further filtered and handled via MS-CleanR by using a minimum blank ratio kept at 0.8 and a maximum relative standard deviation (RSD) adjusted to 100^25^. The maximum mass difference for feature relationship detection and the maximum R_t_ difference were maintained at 0.005 Da and 0.025 min, respectively. Pearson correlation links were considered only for correlations of at least 0.8, with statistical significance defined at a *p‑value* threshold of 0.05.

In each cluster, two peaks were retained for database search, incorporating the most abundant and the most connected feature. Features, retained by MSCleaneR, were annotated with version 3.52 of MS-FINDER^26^. The tolerances of MS1 and MS2 were maintained at 5 and 15 ppm, respectively, with a 1% relative abundance cut-off. By using atoms of C, H, O, P, S, and N, the formula finder was processed with 20% isotopic ratio tolerance.

Using its integrated generic databases, MS-FINDER interrogated the filtered compounds by matching their exact masses and fragmentation patterns (i.e., UNPD, COCONUT, HMDB, FooDB, CHEBI, and LipidMaps). Consequently, the annotated metabolites were assigned a Level 2 confidence (level 2: probable structure with library/bibliography spectral data match according to the Schymanski et al. (2014) framework^27^.

### Multivariate data analyses

Approaches for feature filtration were conducted to keep the abundant and differential metabolites^28–30^. Features showing absolute fold change ≥ 2 and adjusted P (FDR) ≤ 0.05 versus blank samples were retained. For statistical assessment and multivariate data detection and filtration, Metaboanalyst 5.0 and SIMCA 15.0.2 (Sartorius Stedim Data Analytics AB, Sweden) were adopted^31^.

OPLSDA-S-plot features with P > |50| and P correlation > |0.9| were kept together with major compounds with total ion chromatogram (TIC) normalized max abundance not exceeding 2. The chosen features were manually annotated via Sirius and Mass Hunter^32^.

The correlation between the antioxidant activity and the LC-MS dataset of the putatively annotated metabolites was demonstrated by conducting partial least squares (PLS) analysis. Hence, the variable importance in the projection (VIP) values from the PLS analysis were utilized to determine the metabolites responsible for the activity.

The PLS model was validated by permutation tests (*n* = 200) and regression analysis. The model fitness parameters (goodness of fit: R^2^_Y_; goodness of prediction: Q^2^; and cross-validation: CV-ANOVA, *p*-value < 0.01) were also determined (**Fig. S33–S36**).

### Antioxidant activity

Regarding antioxidant analysis, extracts of *S. trilobata* flowerheads and leaves were dissolved in methanol at various concentrations (31.25, 62.5, 125, 250, 500, 1000 µg/mL) for the in vitro DPPH and ABTS assays to calculate the half maximal inhibitory concentration (IC_50_). Moreover, the respective dried extracts were dissolved separately in DMSO at 1 mg/mL to estimate micromole Trolox equivalent per mg sample for the in vitro FRAP assay. The antioxidant assays were conducted using the plate reader Tecan Infinite_F50_.

#### DPPH assay

Radical scavenging activities of *S. trilobata* flowerheads and leaves were assessed using the DPPH assay by calculating the IC_50_^33^. A concentration of 0.04 g% DPPH was prepared in methanol. Then, 20 µL of the diluted concentrations of organs’ extracts were added to 200 µL of 2, 2-diphenyl-1-picrylhydrazyl (DPPH) in a 96-well plate. The absorbance was estimated at 593 nm after a 30-minute incubation period in the dark at room temperature. Ascorbic acid was used as the standard with concentrations (31.25–1000 µg/mL). A blank was prepared in parallel. The samples were tested in triplicate.

#### FRAP assay

The assay was conducted according to the method of Benzie and Strain, with minor changes to be performed in microplates. 190 µL from the freshly prepared 2,4,6-tris(2-pyridyl)-s-triazine reagent (TPTZ) was incorporated with 10 µL of the samples (1 mg/mL) in the 96-well plate in triplicate, and the microplate was kept at room temperature for 30 min protected from the light. The resulting blue color was measured at 593 nm. Additionally, the ferric reducing antioxidant power was determined in µM of TE^34^.

#### ABTS assay

The ABTS assay was conducted according to El-Shiekh et al., with slight modifications^35^. The reagent was dissolved in distilled water (192 mg/50 mL) and mixed with 17 µL of 140 mM potassium persulfate and incubated in a dark place for 16 h. Then, 1mL of the mixture was diluted with methanol to obtain 50 mL of the final ABTS reagent. In a 96-well plate, 190 µL of the freshly prepared ABTS reagent was mixed with 10 µL of the tested extracts with different concentrations (31.25–1000 µg/mL). The reaction was left at room temperature in a dark place for 30 min. A blank was prepared in parallel. The intensity of ABTS color was recorded at 690 nm at the end of the incubation time. Ascorbic acid was utilized as a standard.

#### Statistical analysis

All antioxidant results were acquired through triplicate experiments and expressed as mean ± SD. One-way ANOVA analysis was utilized (GraphPad Prism 8.0, USA). Differences were deemed significant at *p*-value ≤ 0.05.

## Conclusion

Using UPLC-ESI-QTOF-MS/MS and multivariate chemometrics, this study provided a comparative metabolomic and antioxidant profile of *S. trilobata* flowerheads and leaves. A total of 86 distinct metabolites were putatively annotated, including 18 compounds reported for the first time in *S. trilobata*, belonging to the flavonoid, phenolic acid, and terpenoid classes. This integrated study demonstrates that flowerheads have significantly higher in vitro antioxidant capacity than leaves. It also identifies several flavonoids (notably eriodictyol and myricetin derivatives), dicaffeoyl-quinic acids, and certain pyroglutamic acid derivatives that are strongly correlated with the observed activity and likely contribute to it, supporting a biochemical basis for the plant’s traditional uses. These correlations suggest, but do not prove, mechanistic roles; further isolation and in vivo/cellular validation are warranted.

These conclusions should, however, be interpreted in light of some limitations. The study used a small number of biological replicates and samples were collected under limited conditions. Therefore, environmental and population-level phytochemical variability remain uncharacterized.

To address these gaps, priority next steps should include isolation and full structural elucidation of the top-ranked metabolites to confirm their identities; biological validation of antioxidant and cytoprotective effects in cellular assays and in vivo models to assess efficacy, dose–response, bioavailability, synergistic interactions, and safety; and broader sampling across locations and seasons to define phytochemical variability and optimize harvesting and processing. Additionally, once marker compounds are validated, development of targeted quantitative methods would support quality-control standards for standardized extracts or botanical products.

## Supplementary Information

Below is the link to the electronic supplementary material.

Supplementary Material 1

## Data Availability

Data will be made available on request.
