# Supplementary material for: Integrated metabolomics and antioxidant activity assessment of Sphagneticola trilobata (L.) Pruski
Source: Sci Rep. 2026 Jun 13;16:18361. doi: 10.1038/s41598-026-54096-w (PMC13264626; doi:10.1038/s41598-026-54096-w)
Supplement: Supplementary file 1 — Supplementary Material 1 [file 41598_2026_54096_MOESM1_ESM.pdf]

Integrated Metabolomics and Antioxidant Activity Assessment of *Sphagneticola trilobata* (L.) Pruski

Manar T. Ali[a,*,#], Muhammed A. Alsherbiny[a,c, #], Dalia A. Al-Mahdy[a,b], Ahlam M. El Fishawy[a], Asmaa M. Otify[a,*]

[a] *Department of Pharmacognosy, Faculty of Pharmacy, Cairo University, Kasr-El-Ainy, Cairo 11562, Egypt*

[b] *Department of Pharmacognosy, Faculty of Pharmacy, Modern University for Technology and Information (MTI University), Cairo, Egypt*

[c] *NICM Health Research Institute, Western Sydney University, Westmead, NSW, 2145, Australia*

[#] *These authors contributed equally to this work.*

[*] *Corresponding authors: Asmaa M. Otify (asmaa.otify@pharma.cu.edu.eg), Manar T. Ali (manar.tarek@pharma.cu.edu.eg)*

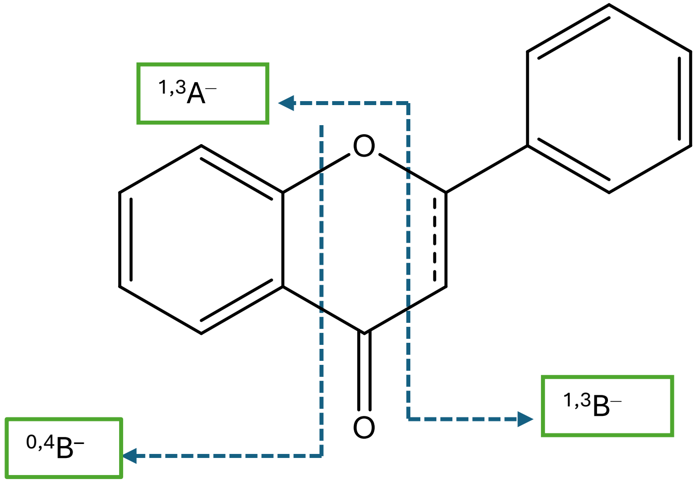

Fig. S**1**: Nomenclature proposed by Ma et.al. (1997) to represent fragment ions resulted from fragmentation of detected flavonoids.

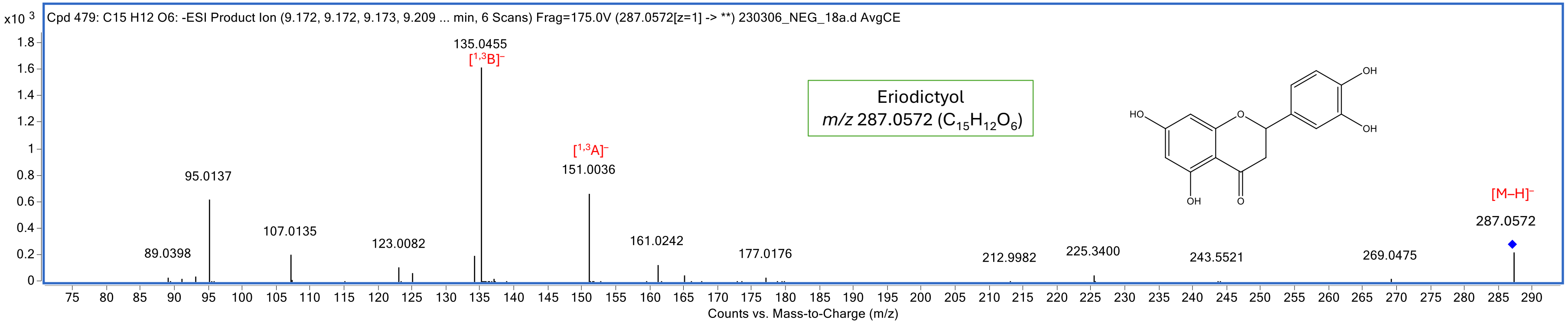

Fig. S2: MS/MS spectrum of metabolite **33** in negative mode.

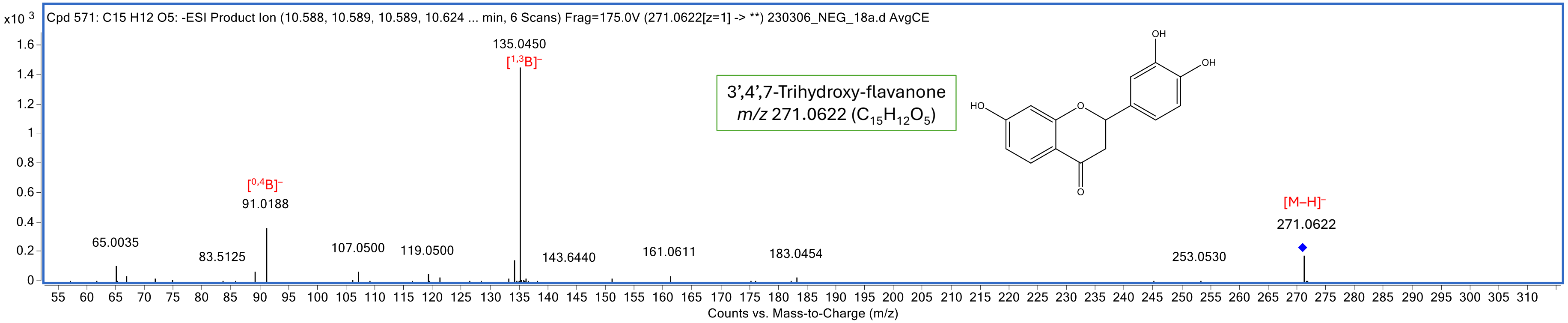

Fig. S3: MS/MS spectrum of metabolite **37** in negative mode.

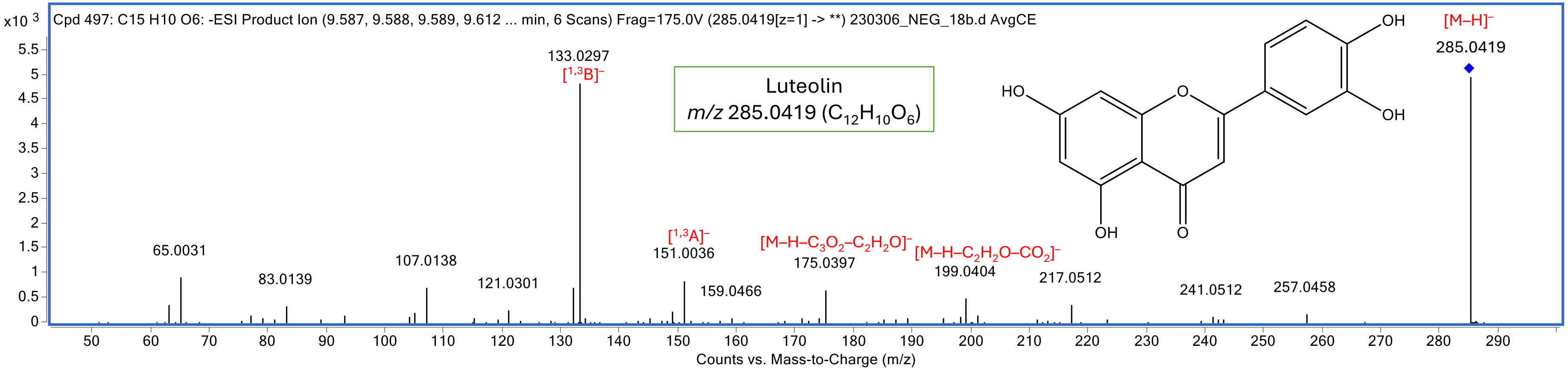

Fig. S4: MS/MS spectrum of metabolite **34** in negative mode.

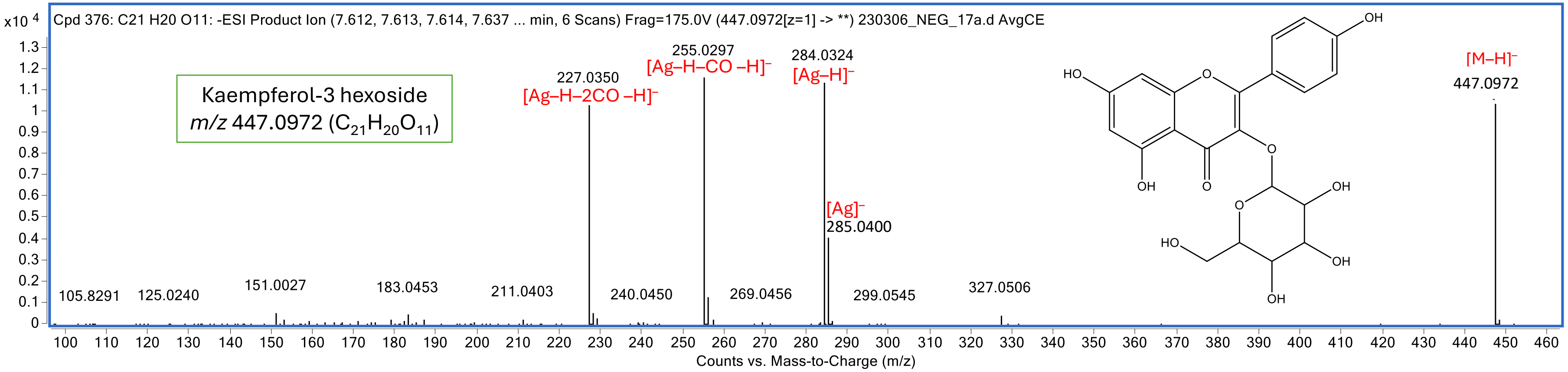

Fig. S5:MS/MS spectrum of metabolite **29** in negative mode.

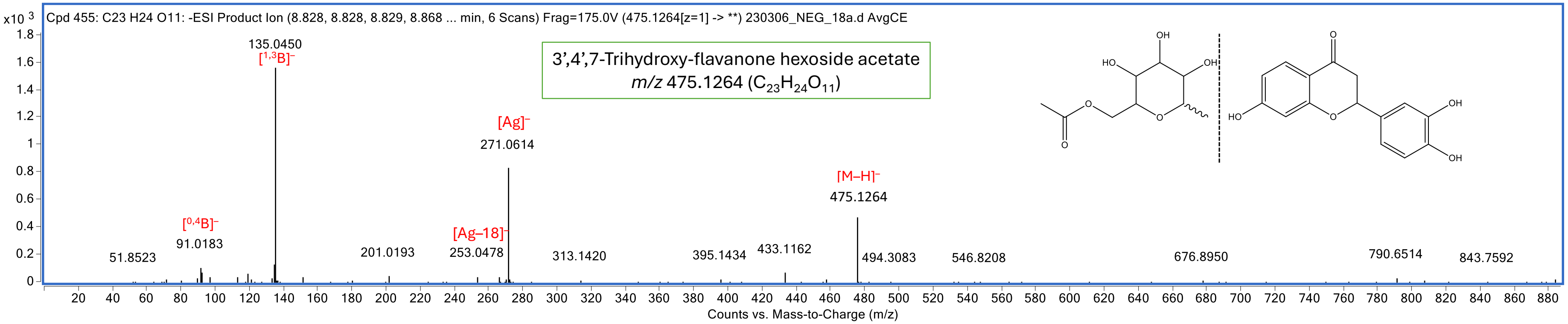

Fig. S6: MS/MS spectrum of metabolite **32** in negative mode.

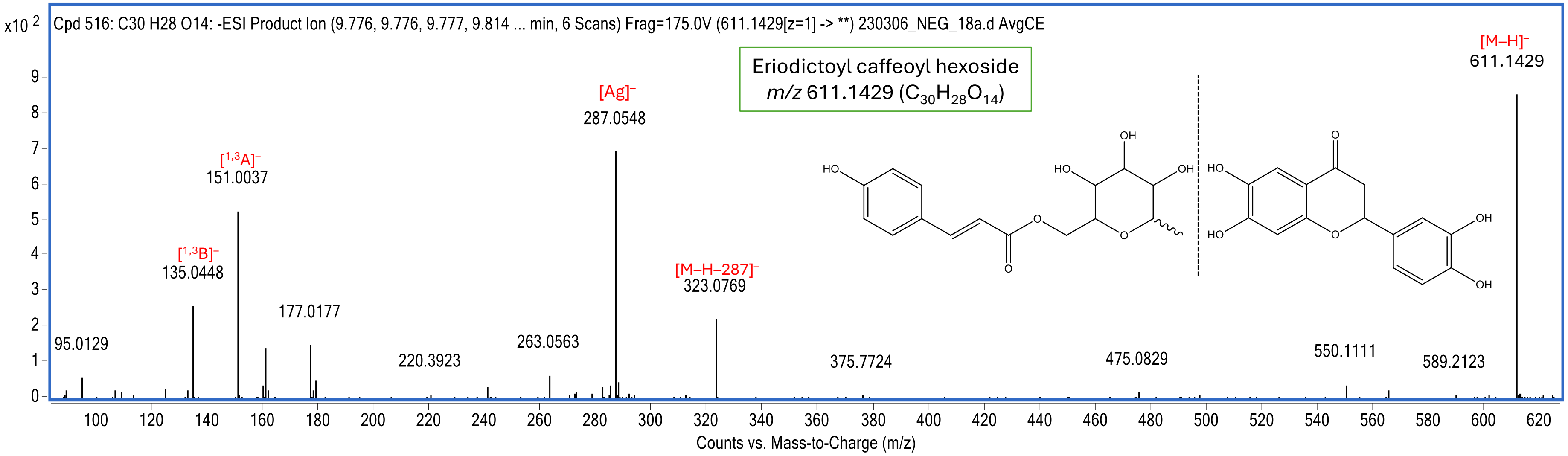

Fig. S7: MS/MS spectrum of metabolite **35** in negative mode.

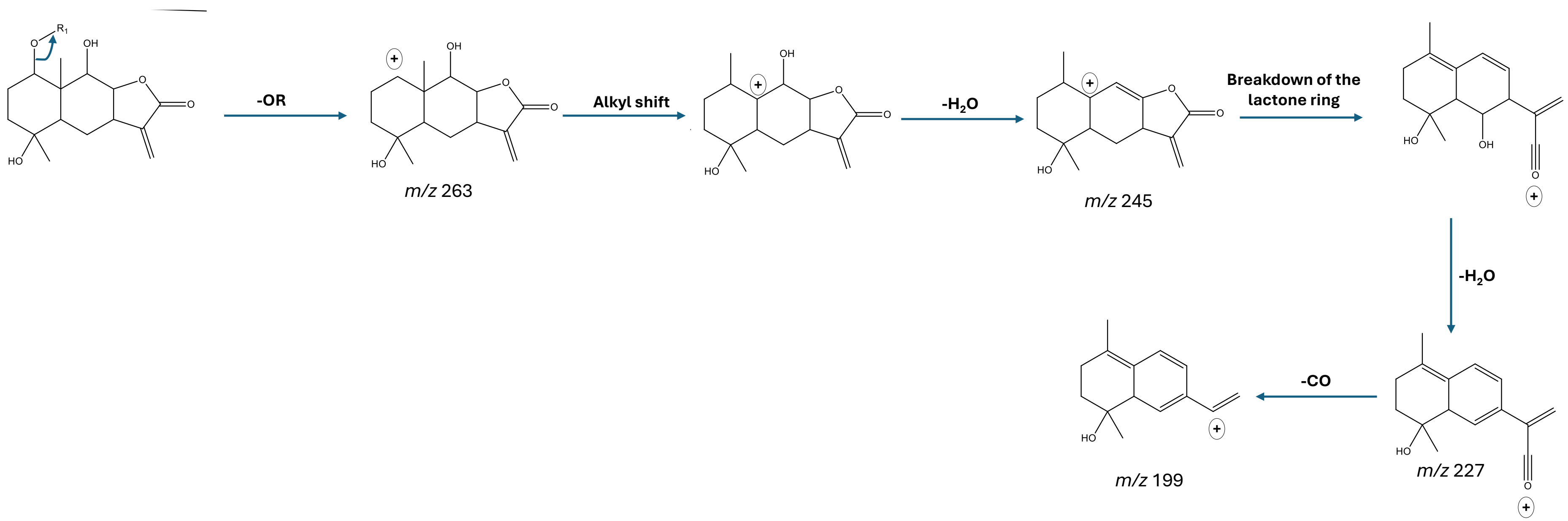

Fig. S8: The scheme of eudesmanolides sequential fragmentation in positive mode.

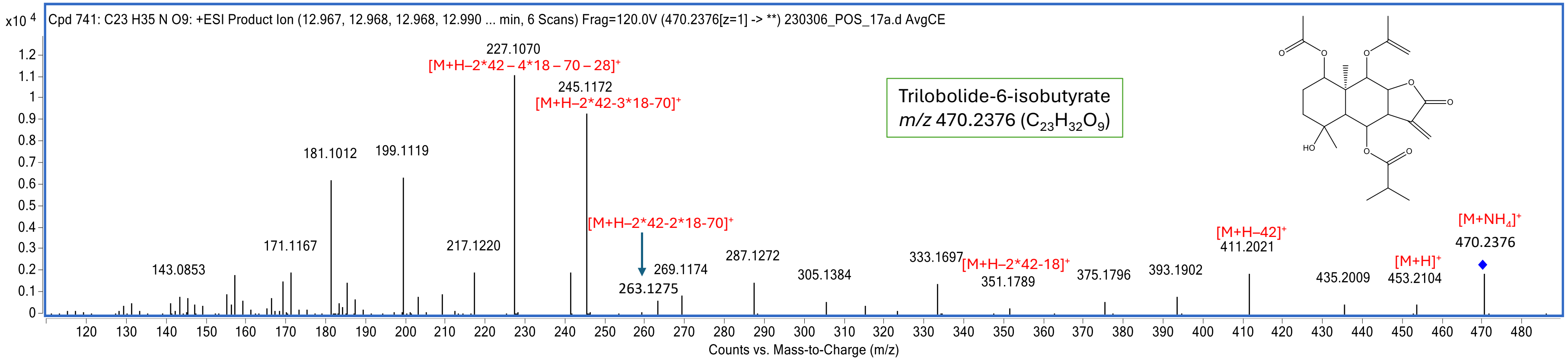

Fig. S9: MS/MS spectrum of metabolite **50** in positive mode.

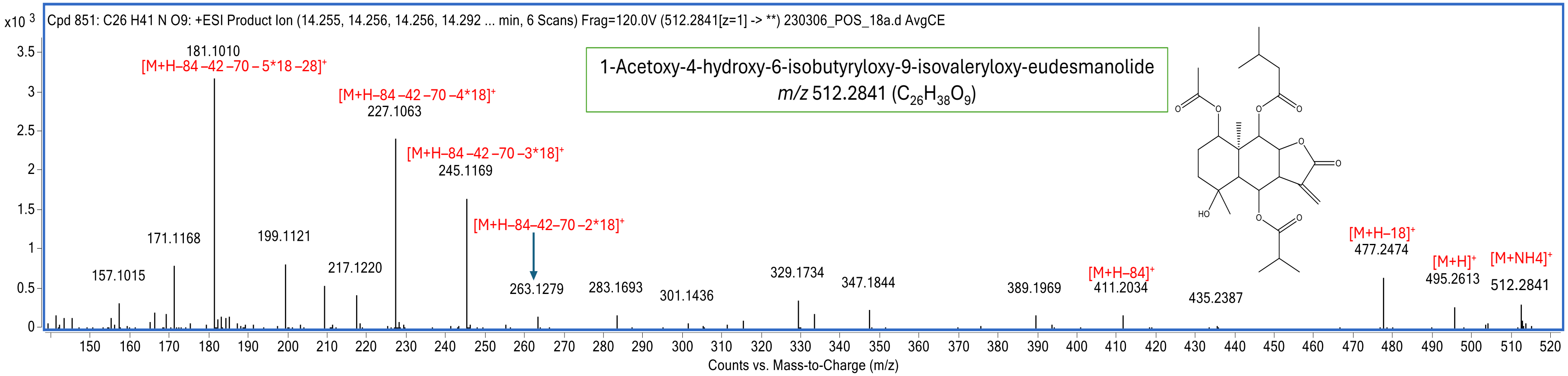

Fig. S10: MS/MS spectrum of metabolite **59** in positive mode.

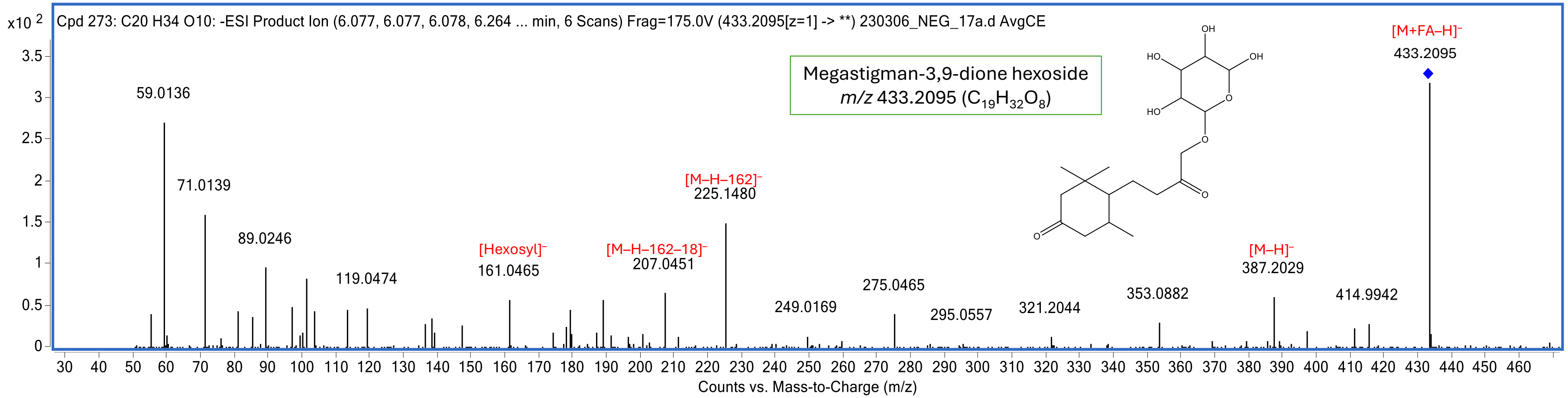

Fig. S11: MS/MS spectrum of metabolite **44** in positive mode.

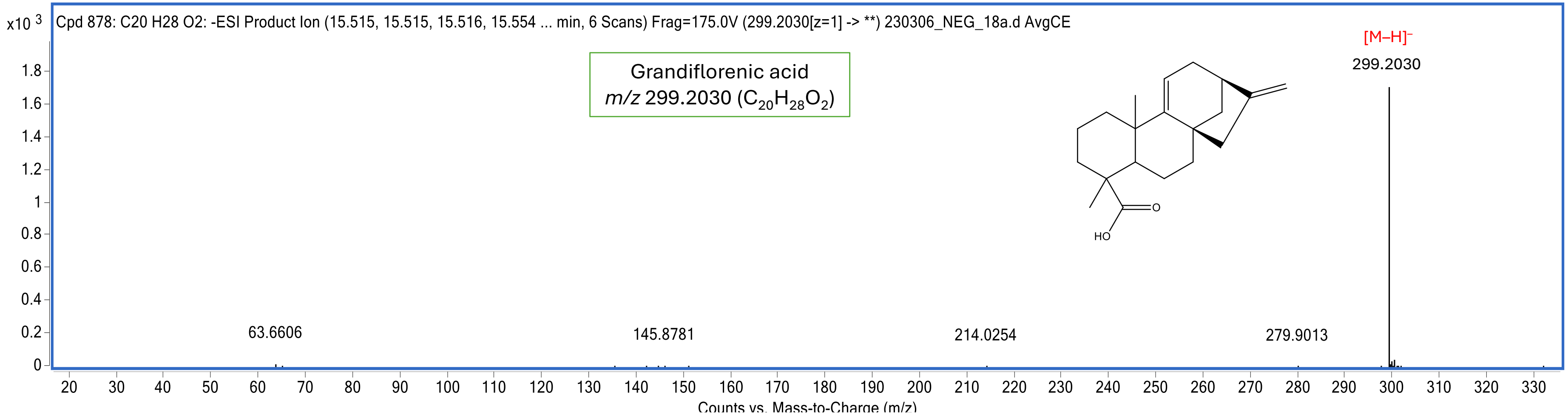

Fig. S12: MS/MS spectrum of metabolite **68** in negative mode.

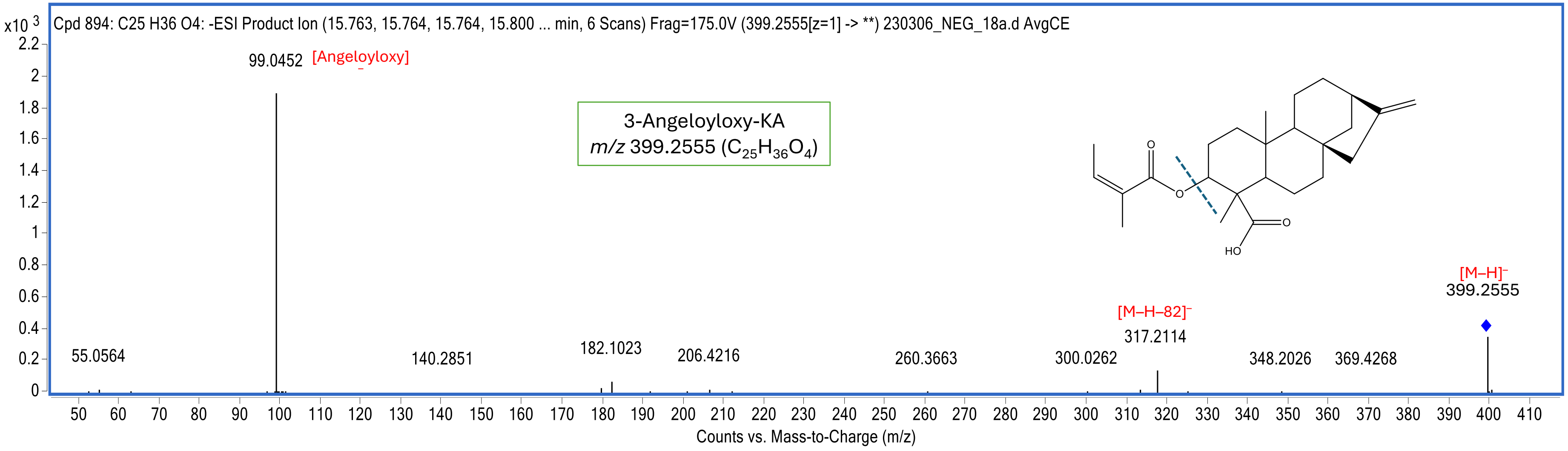

Fig. S13: MS/MS spectrum of metabolite **69** in negative mode.

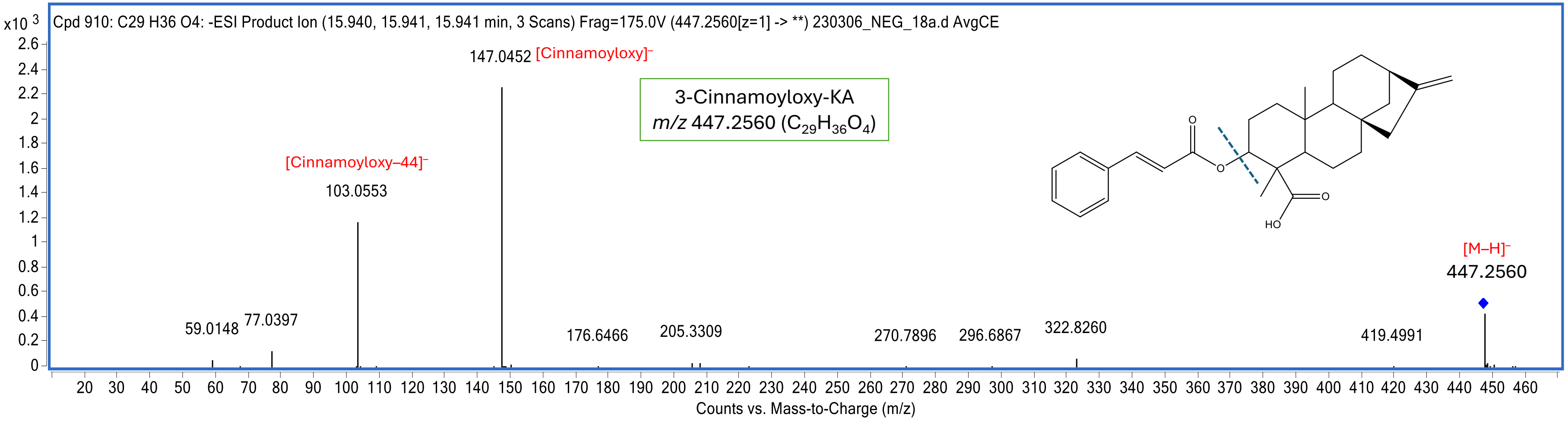

Fig. S14: MS/MS spectrum of metabolite **67** in negative mode.

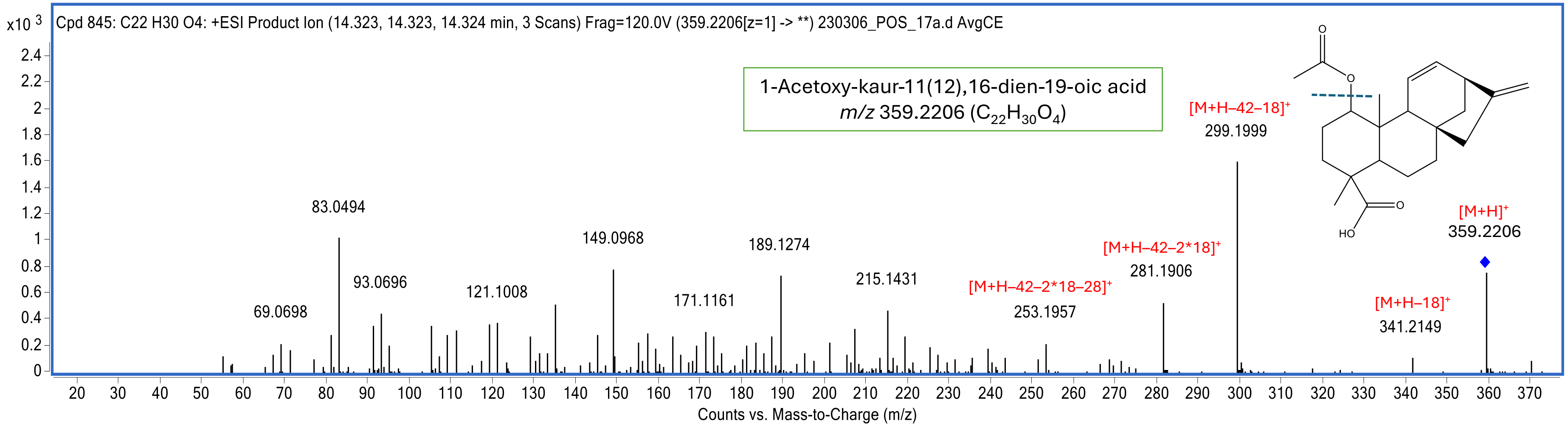

Fig. S15: MS/MS spectrum of metabolite **61** in positive mode.

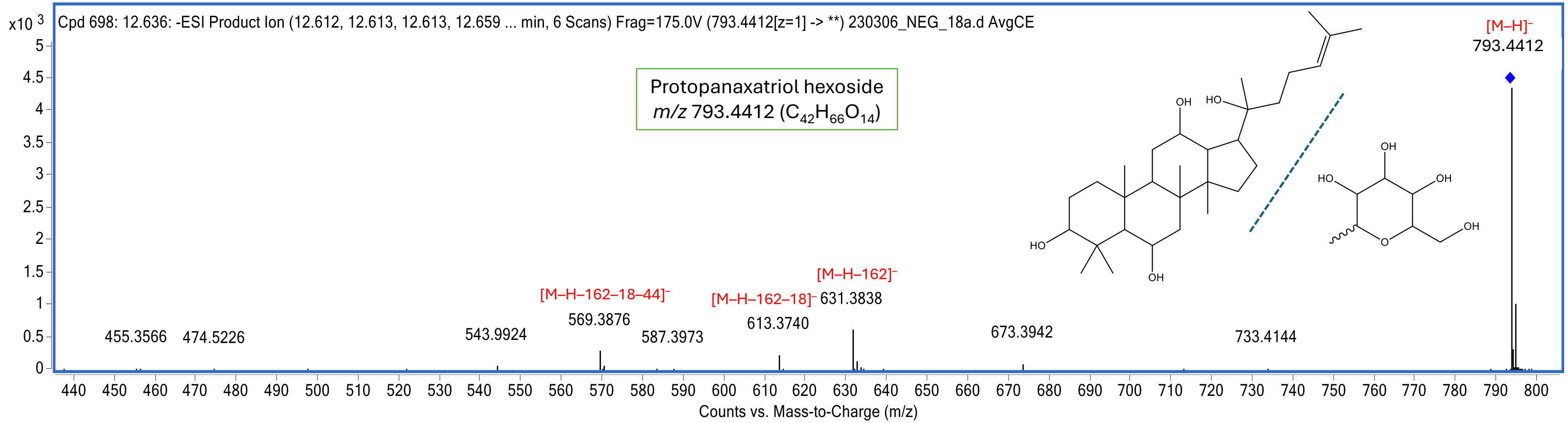

Fig. S16: MS/MS spectrum of metabolite **48** in negative mode.

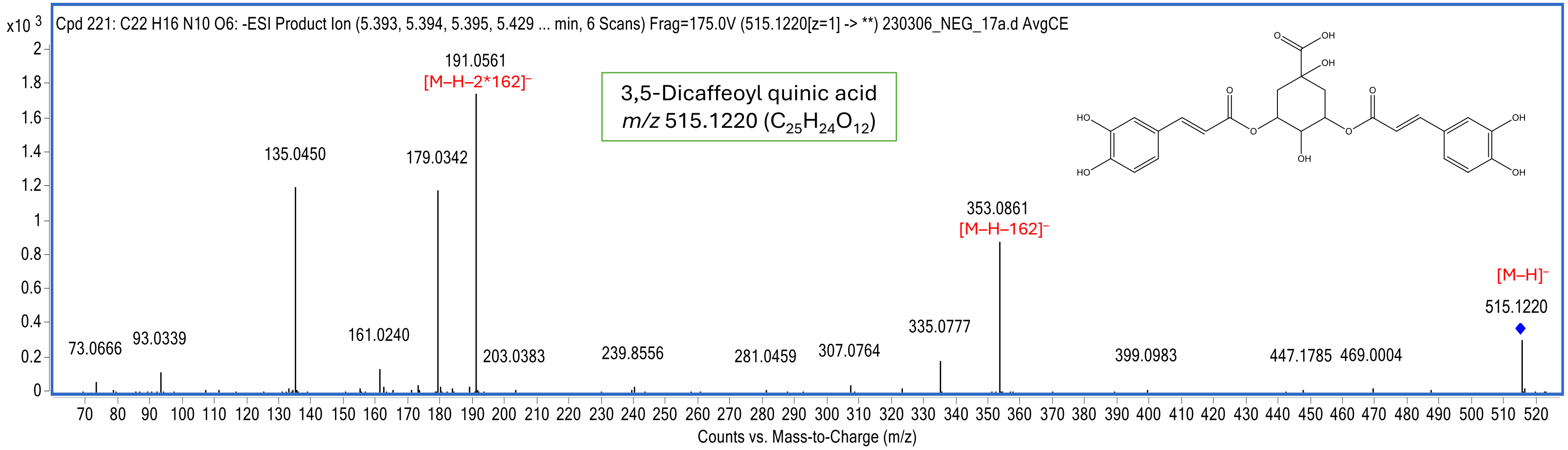

Fig. S17: MS/MS spectrum of metabolite **12** in negative mode.

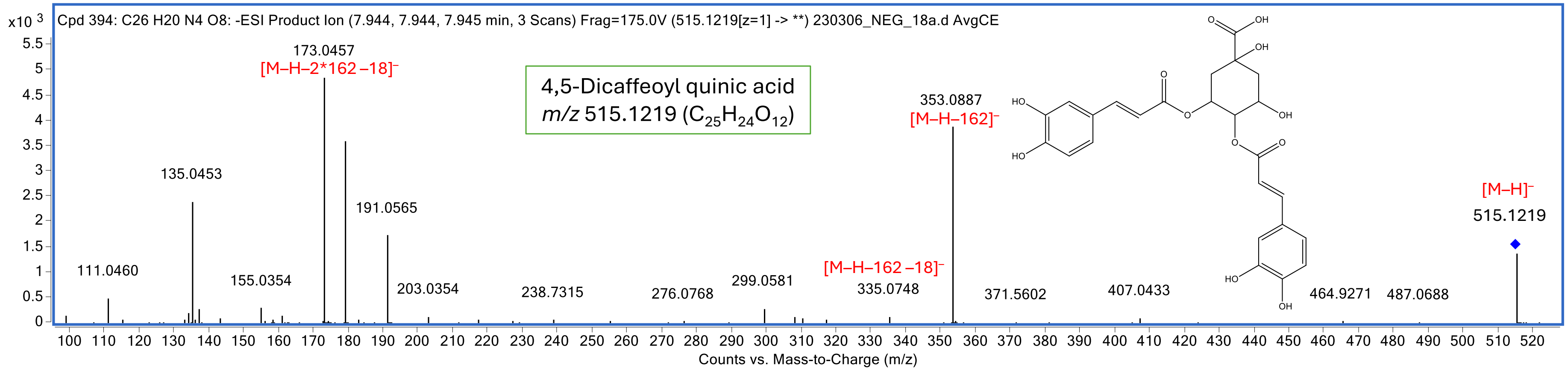

Fig. S18: MS/MS spectrum of metabolite **13** in negative mode.

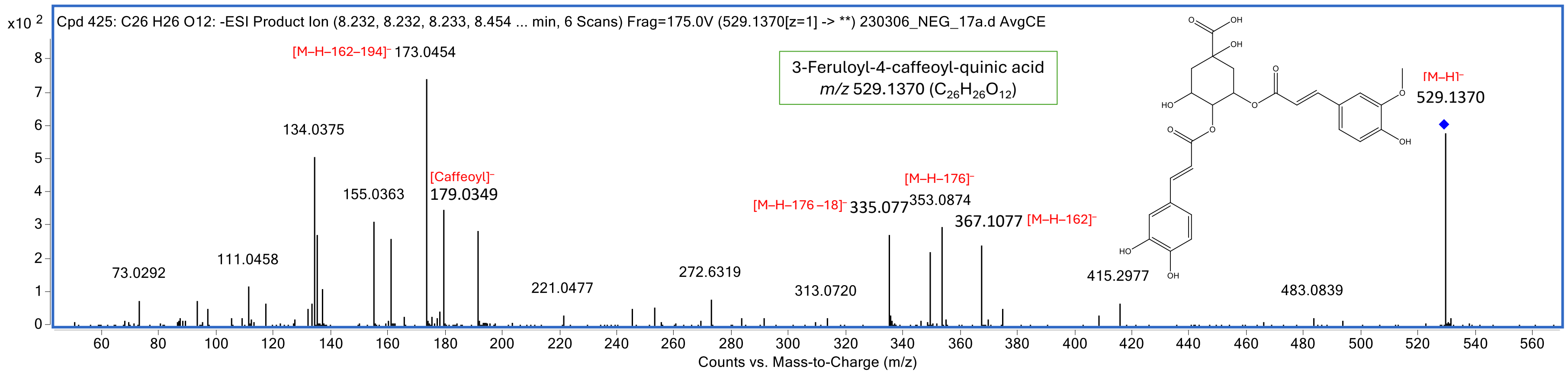

Fig. S19: MS/MS spectrum of metabolite **14** in negative mode.

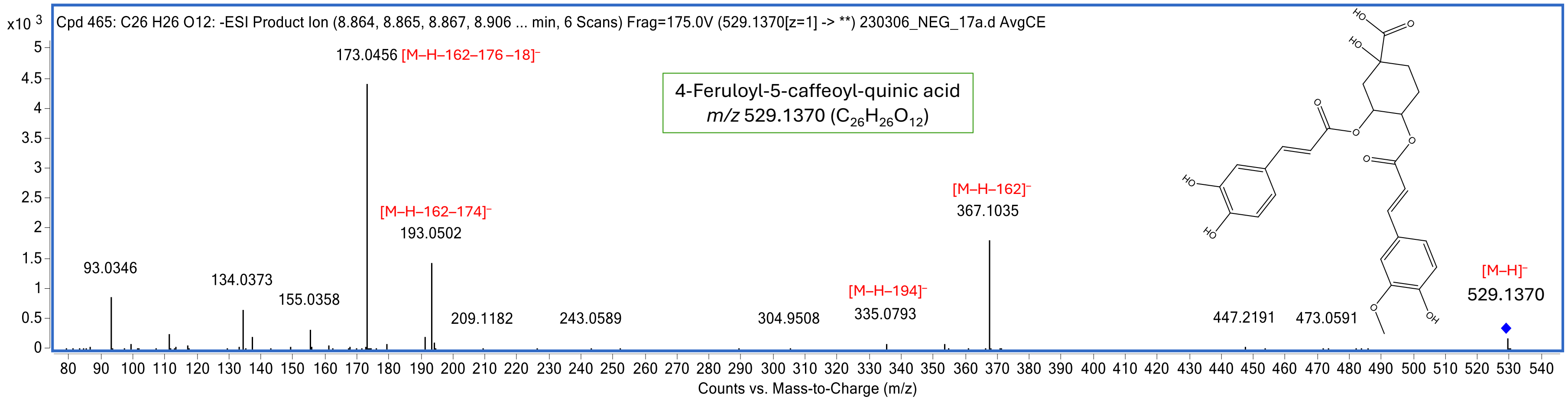

Fig. S20: MS/MS spectrum of metabolite **18** in negative mode.

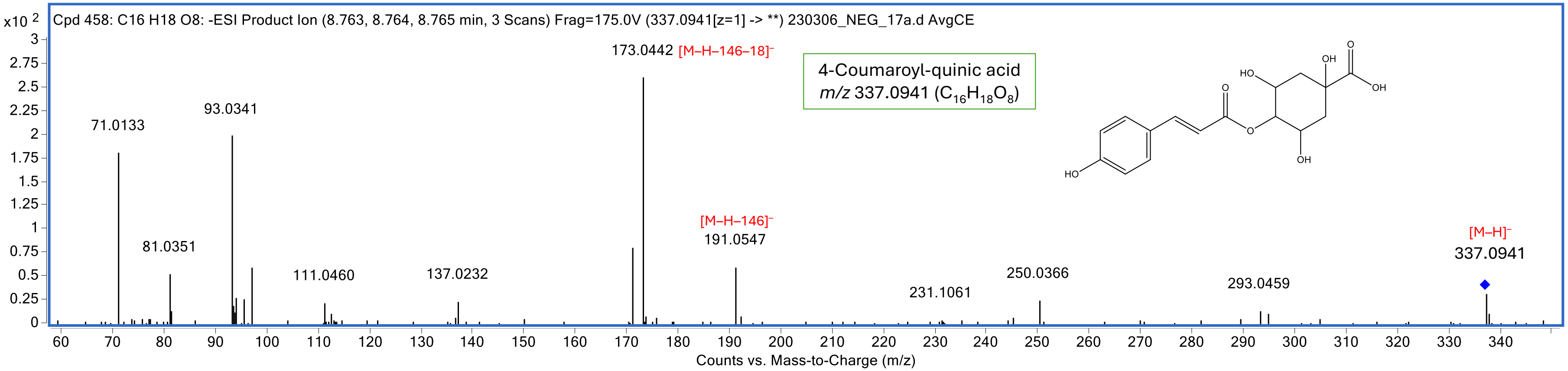

Fig. S21: MS/MS spectrum of metabolite **15** in negative mode.

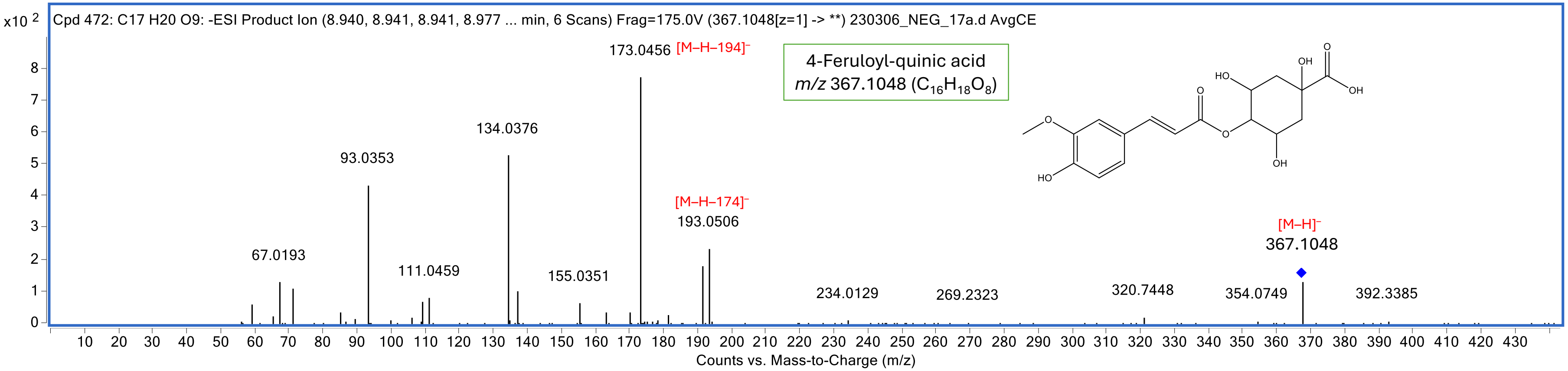

Fig. S22: MS/MS spectrum of metabolite **16** in the negative mode.

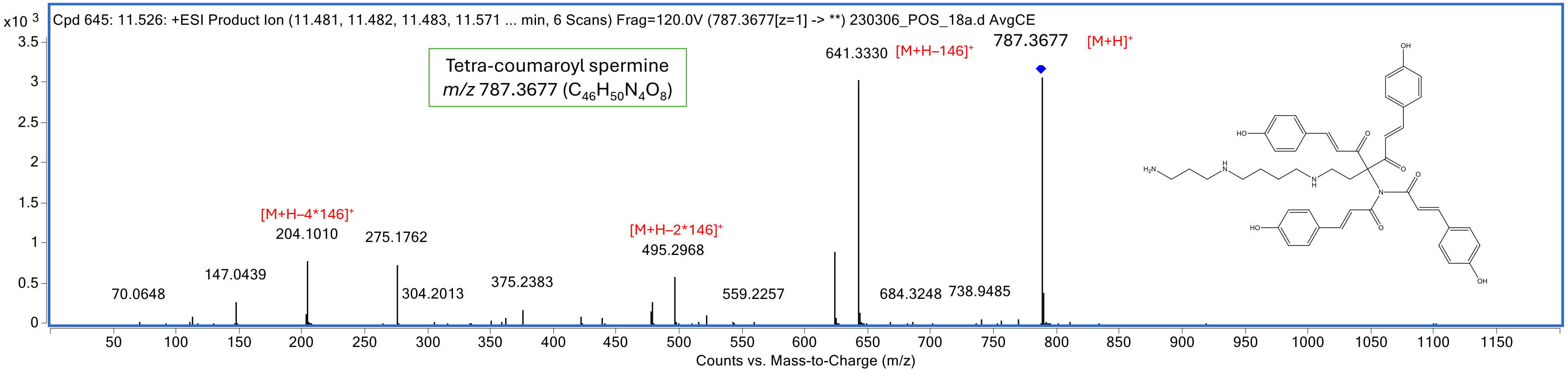

Fig. S23: MS/MS spectrum of metabolite **21** in the positive mode.

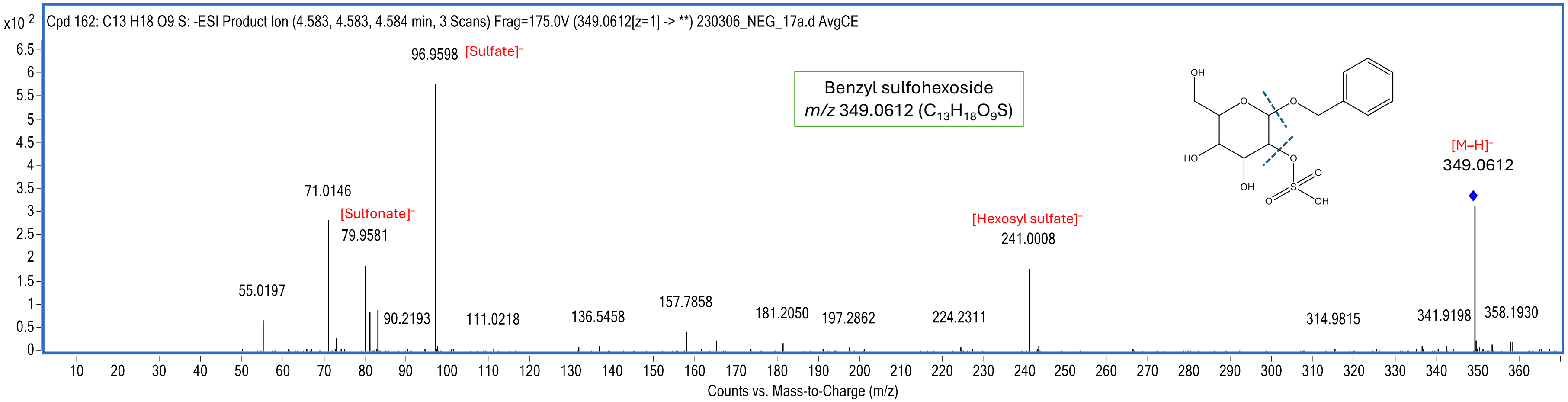

Fig. S24: MS/MS spectrum of metabolite **40** in negative mode.

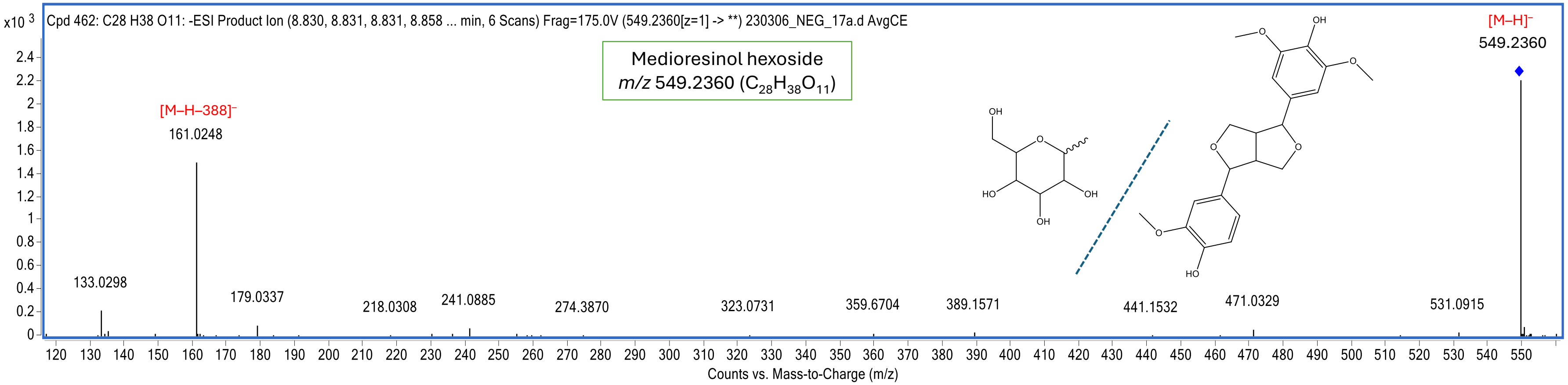

Fig. S25: MS/MS spectrum of metabolite **41** in negative mode.

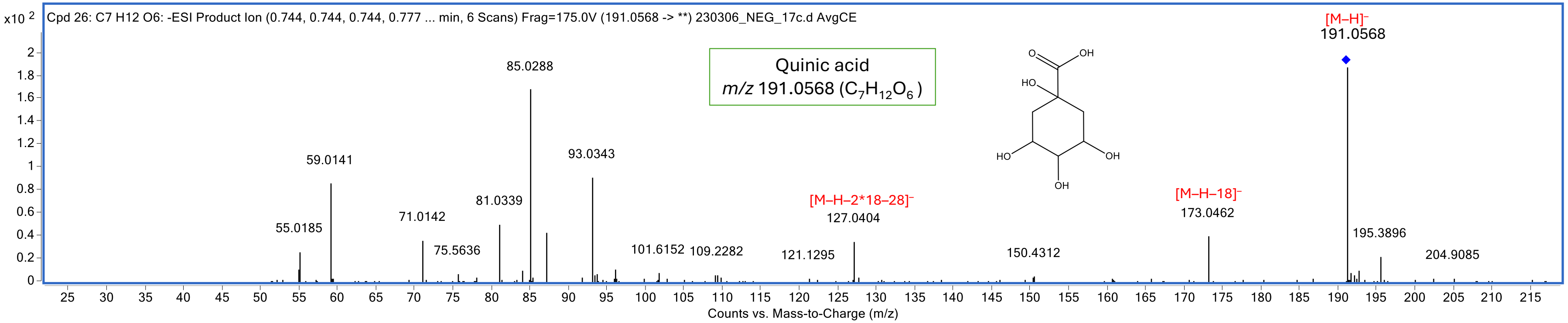

Fig.S26: MS/MS spectrum of metabolite **70** in negative mode.

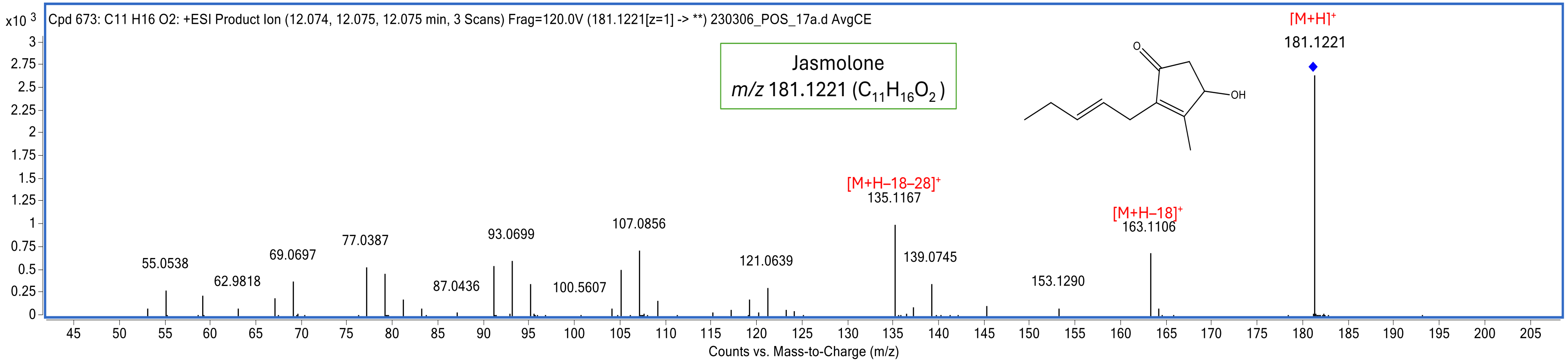

Fig. S27: MS/MS spectrum of metabolite **75** in positive mode.

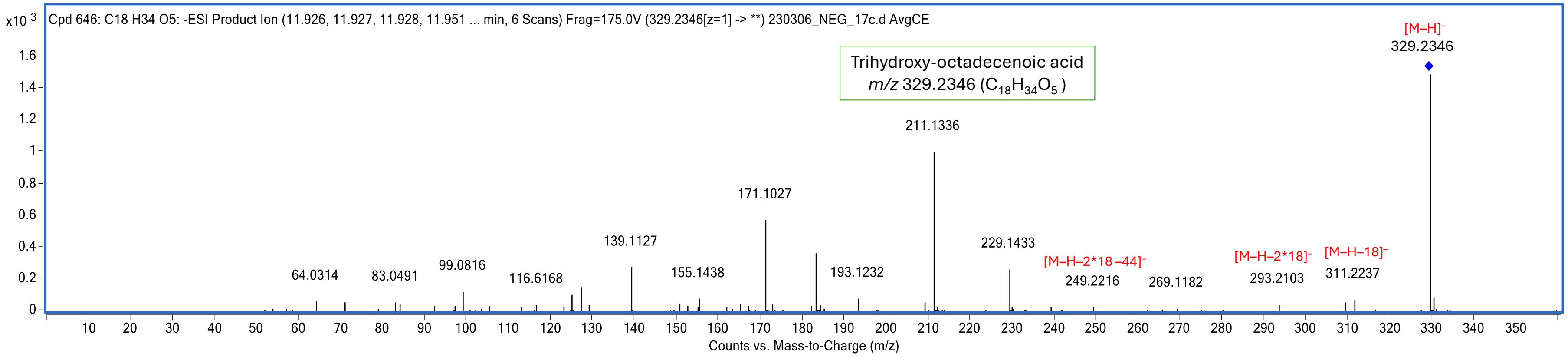

Fig. S28: MS/MS spectrum of metabolite **74** in negative mode.

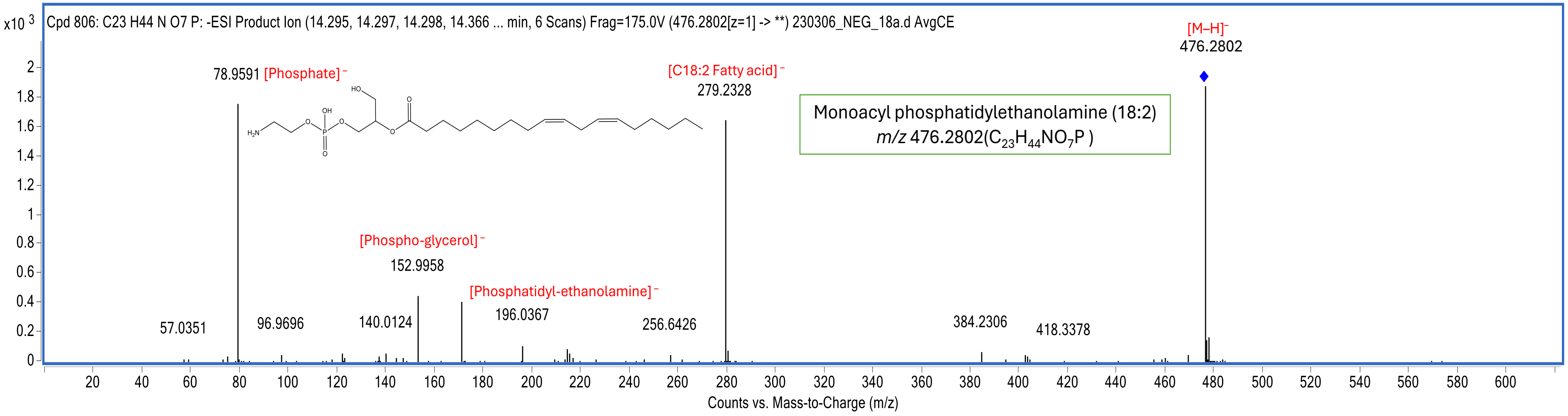

Fig. S29: MS/MS spectrum of metabolite **80** in negative mode.

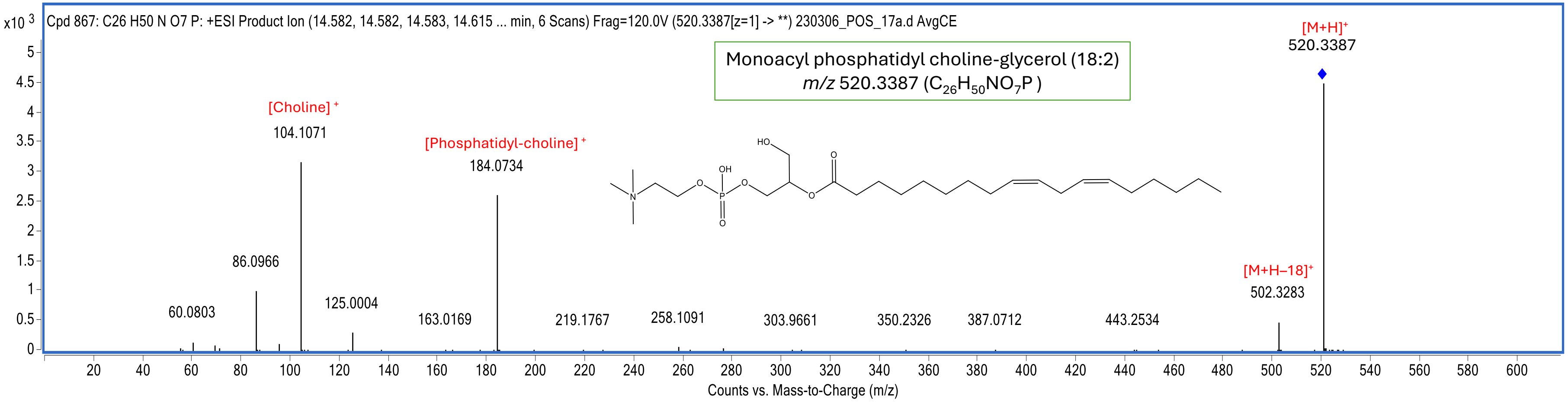

Fig. S30: MS/MS spectrum of metabolite **81** in positive mode.

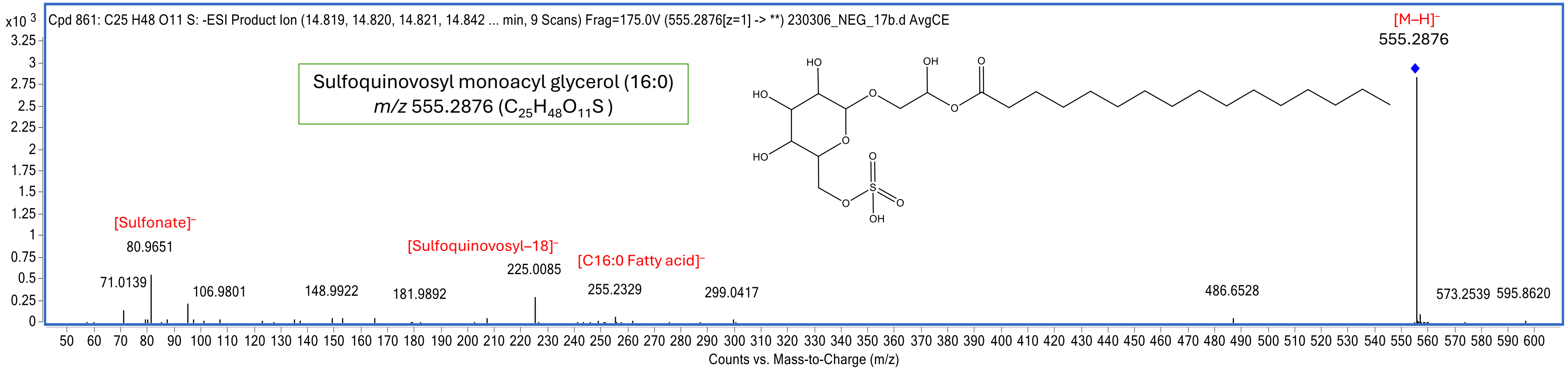

Fig. S31: MS/MS spectrum of metabolite **84** in negative mode.

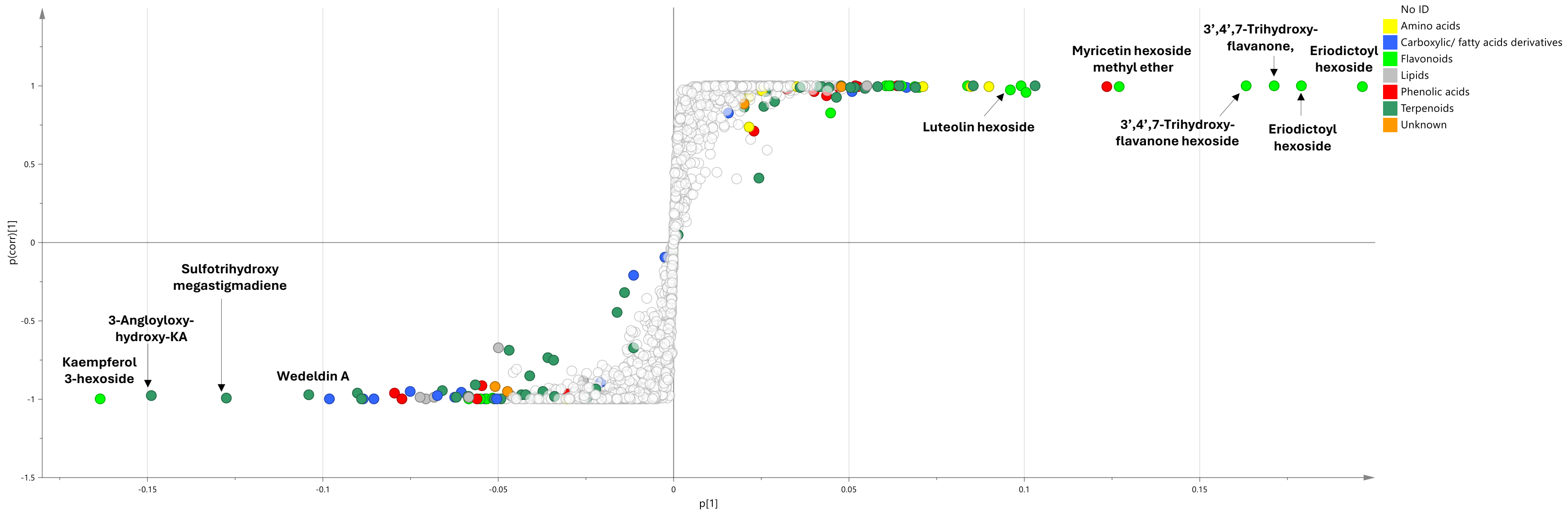

Fig. S32: OPLS showing loading scatter S-Plot derived from UPLC-MS significant 1666 features of *S. trilobata* flowerheads vs leaves. Compounds are colored by chemical classes and were sized according to the average normalized intensity.

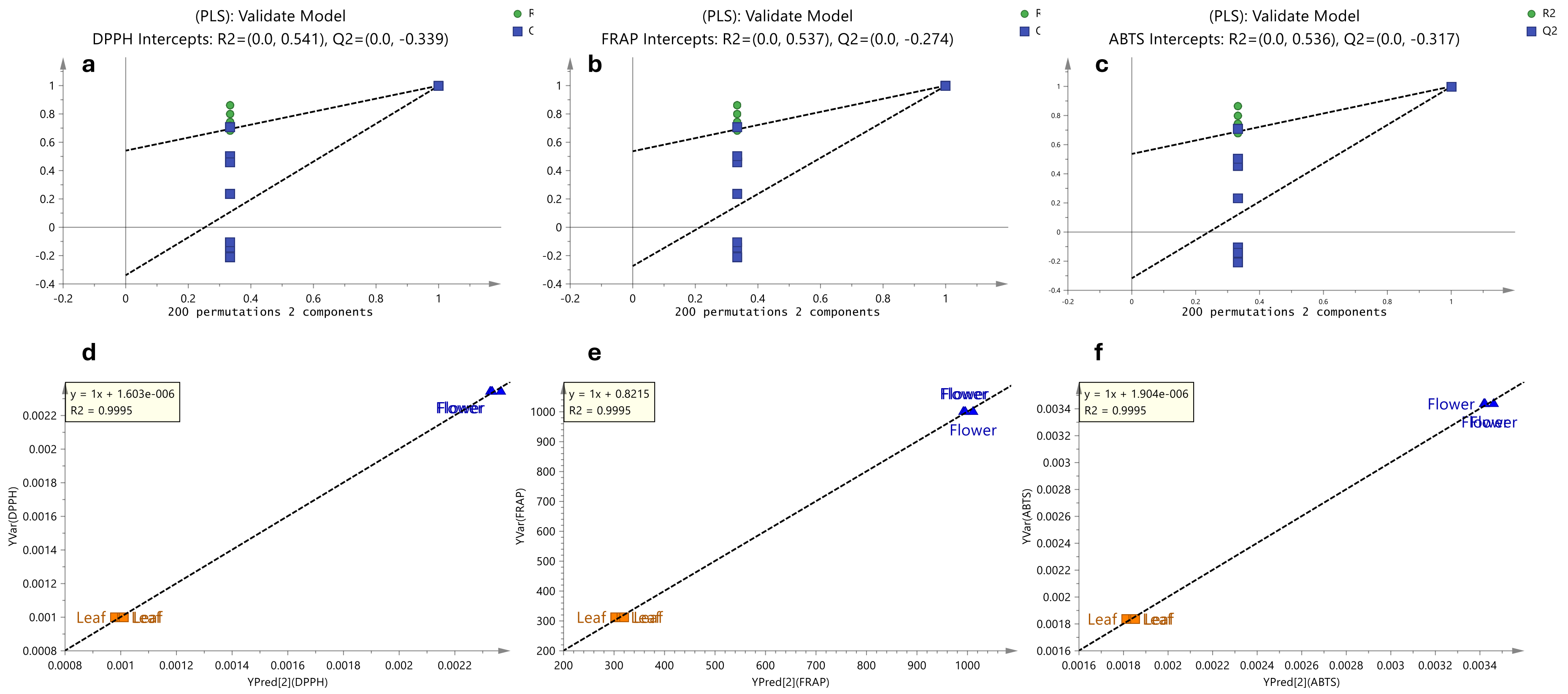

Fig. S33: UPLC-MS based PLS validation parameters: the permutation tests of a. DPPH and b.FRAP antioxidant assays; and PLS derived relationship between observed vs predicted for c. DPPH and d.FRAP antioxidant assays in positive mode.

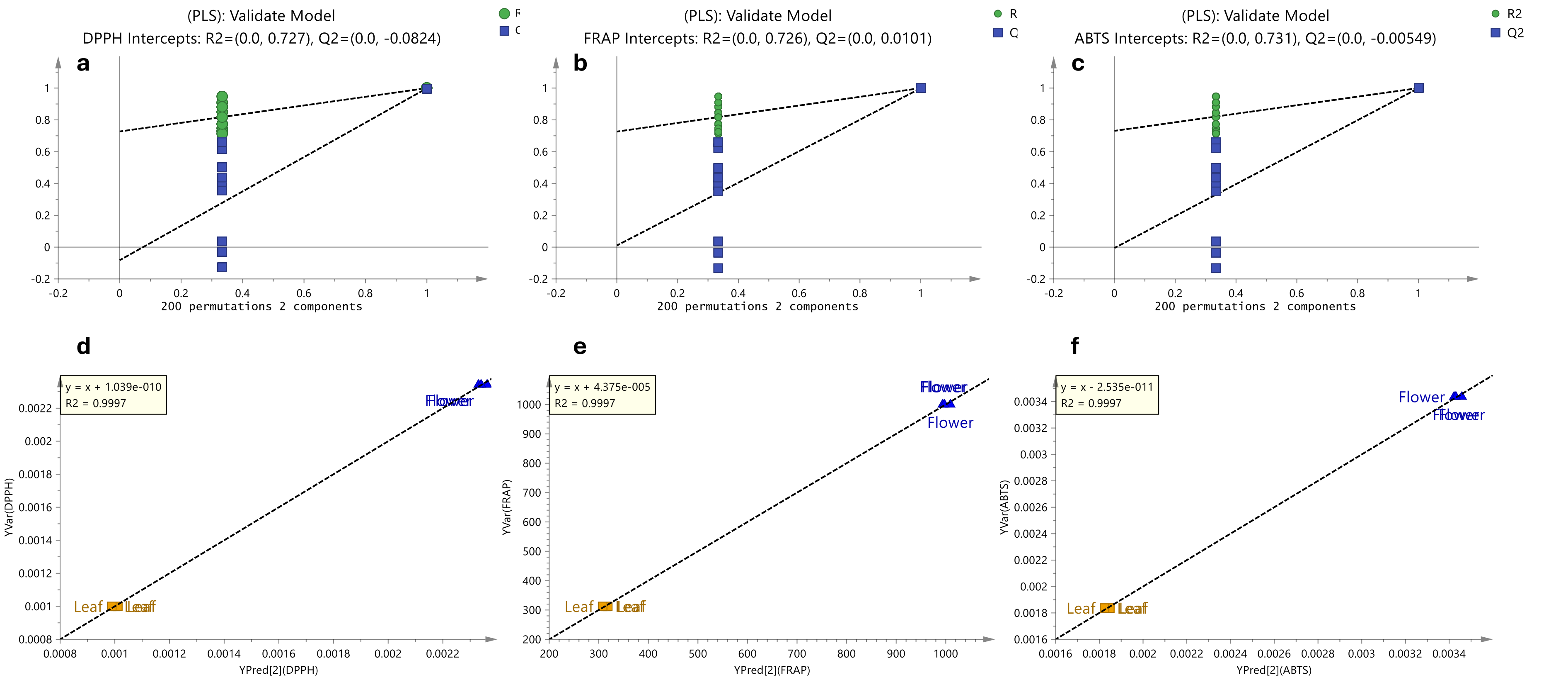

Fig. S34: UPLC-MS based PLS validation parameters: the permutation tests of a. DPPH and b.FRAP antioxidant assays; and PLS derived relationship between observed vs predicted for c. DPPH and d. FRAP antioxidant assays in negative mode.

a

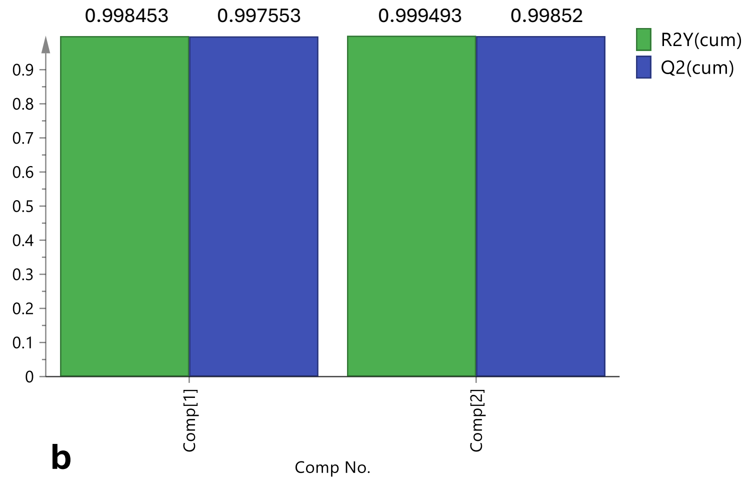

b

M2(Positive)	SS	DF	MS	F	p	SD
**DPPH**						
**Total corr.**	5	5	1			1
**Regression**	4.99279	3	1.66426	461.631	0.00216233	1.29006
**Residual**	0.00721036	2	0.00360518			0.0600431
**FRAP**						
**Total corr.**	5	5	1			1
**Regression**	4.99279	3	1.66426	461.631	0.00216233	1.29006
**Residual**	0.00721036	2	0.00360518			0.0600431
**ABTS**						
**Total corr.**	5	5	1			1
**Regression**	4.99279	3	1.66426	461.631	0.00216233	1.29006
**Residual**	0.00721036	2	0.00360518			0.0600431

Fig. S35: UPLC-MS based PLS optimization and validation parameters. **a.** The diagnostic metrics R^2_Y and Q^2 as function of number of principal components in positive mode for antioxidant activities. **b.** Cross-validation CV-ANOVA to assess for model statistical significance for antioxidant activities in positive mode.

a

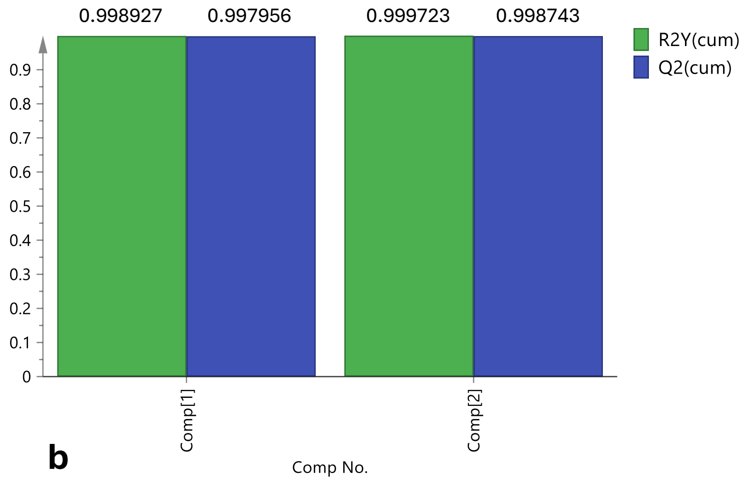

b

M1(Negative)	SS	DF	MS	F	p	SD
**DPPH**						
**Total corr.**	5	5	1			1
**Regression**	4.99196	3	1.66399	413.827	0.00241161	1.28996
**Residual**	0.00804193	2	0.00402097			0.0634111
**FRAP**						
**Total corr.**	5	5	1			1
**Regression**	4.99196	3	1.66399	413.827	0.00241161	1.28996
**Residual**	0.00804193	2	0.00402097			0.0634111
**ABTS**						
**Total corr.**	5	5	1			1
**Regression**	4.99196	3	1.66399	413.827	0.00241161	1.28996
**Residual**	0.00804193	2	0.00402097			0.0634111

.

Fig. S36: UPLC-MS based PLS optimization and validation parameters. **a.** The diagnostic metrics R^2_Y and Q^2 as function of number of principal components in negative mode for antioxidant activities. **b.** Cross-validation CV-ANOVA to assess for model statistical significance for antioxidant activities in negative mode.
